# Fresh Impetus in the Chemistry of Calcium Peroxides

**DOI:** 10.1021/jacs.4c00906

**Published:** 2024-06-07

**Authors:** Arkadiusz Kornowicz, Tomasz Pietrzak, Krzesimir Korona, Michał Terlecki, Iwona Justyniak, Adam Kubas, Janusz Lewiński

**Affiliations:** † Institute of Physical Chemistry, Polish Academy of Sciences, Kasprzaka 44/52, 01-224 Warsaw, Poland; ‡ Faculty of Chemistry, Warsaw University of Technology, Noakowskiego 3, 00-664 Warsaw, Poland

## Abstract

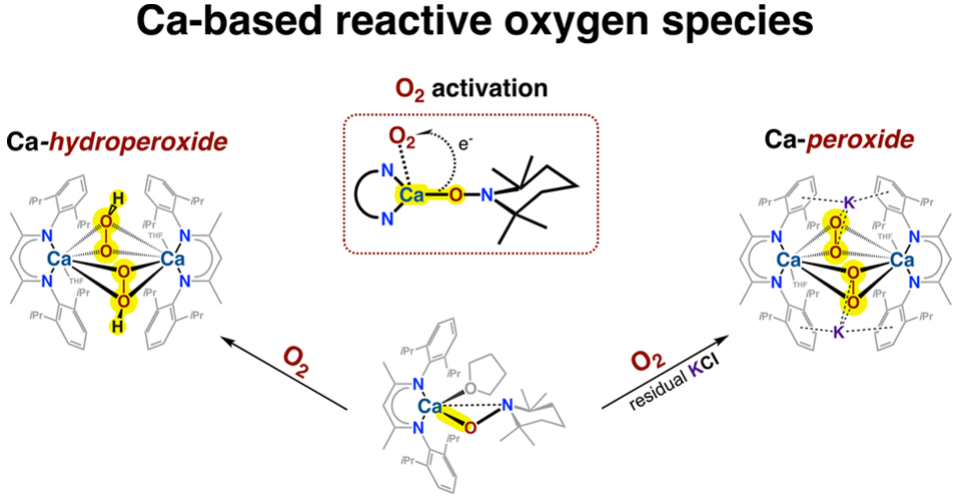

Redox-inactive metal ions are essential in modulating the reactivity of various oxygen-containing metal complexes and metalloenzymes, including photosystem II (PSII). The heart of this unique membrane–protein complex comprises the Mn_4_CaO_5_ cluster, in which the Ca^2+^ ion acts as a critical cofactor in the splitting of water in PSII. However, there is still a lack of studies involving Ca-based reactive oxygen species (ROS) systems, and the exact nature of the interaction between the Ca^2+^ center and ROS in PSII still generates intense debate. Here, harnessing a novel Ca-TEMPO complex supported by the β-diketiminate ligand to control the activation of O_2_, we report the isolation and structural characterization of hitherto elusive Ca peroxides, a homometallic Ca hydroperoxide and a heterometallic Ca/K peroxide. Our studies indicate that the presence of K^+^ cations is a key factor controlling the outcome of the oxygenation reaction of the model Ca-TEMPO complex. Combining experimental observations with computational investigations, we also propose a mechanistic rationalization for the reaction outcomes. The designed approach demonstrates metal-TEMPO complexes as a versatile platform for O_2_ activation and advances the understanding of Ca/ROS systems.

## Introduction

**Scheme 1:**
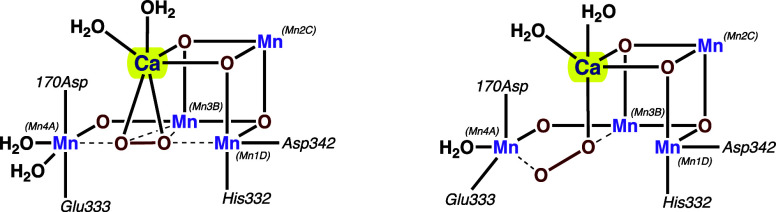
O_2_-Evolving S_4_ Transition State Models in OEC Proposed by Suga et al. (left)^22^ and Messinger and co-workers (right).^28^

Redox-inactive metal ions, including Ca^2+^ cations, are essential in modulating the reactivity of various oxygen-containing metal complexes and metalloenzymes, e.g., the reactivity of transition metal-based reactive oxygen species (ROS)^1−7^ or water oxidation into dioxygen catalyzed by oxygen-evolving complex (OEC) in photosystem II (PSII).^8−13^ For example, the core of PSII consists of the Mn_4_CaO_5_ cluster,^14−17^ and decades of ongoing experimental and theoretical studies have focused on the factors controlling the chemistry of this unique CaMn_4_ assembly during splitting water to dioxygen in the catalytic Kok cycle.^18−28^ The mechanism of O–O bond formation in a short-living S_4_ state (Scheme 1) of the Kok cycle still generates an intense debate, and the reports that have been published so far suggest that the redox inactive Lewis-acidic Ca ion is essential for reactive intermediate stabilization, but its exact role is yet to be eluded.^22,28−34^ Undoubtedly, a detailed understanding of how the Ca^2+^ site facilitates O_2_ formation requires a clear picture of the interaction between the Ca^2+^ center and active oxygen species. Using synthetic structural models of the OEC is an efficient way to understand the processes driven by nature, and, generally, two approaches have been used to elucidate the role of Mn and Ca centers:^35^ (i) the modeling reactivity of predesigned heterometallic Mn/Ca clusters^11,12,29,33,36−39^ and (ii) various cuboidal Mn_*x*_O_*y*_ clusters.^40−47^ However, despite recent advances in modeling the OEC, there is still a lack of studies on Ca-based ROS systems. Herein, we report a unique reactivity of a model Ca-TEMPO (TEMPO = (2,2,6,6-Tetramethylpiperidin-1-yl)oxyl) complex toward O_2_, leading to unprecedented Ca-hydroperoxide and heterometallic Ca/K peroxide species. The observed reactivity of Ca/ROS system relates to the postulated S_4_ transition state in the water splitting process, mediated by the heterometallic Mn_4_CaO_5_ cluster in the PSII system (Scheme 1).^16,22,28^ The isolation of Ca-centered ROS has hitherto eluded the skills of experimentalists, and thus, the presented studies might foster a broader discussion on the role of a Ca^2+^ site in the OEC.

**Scheme 2:**
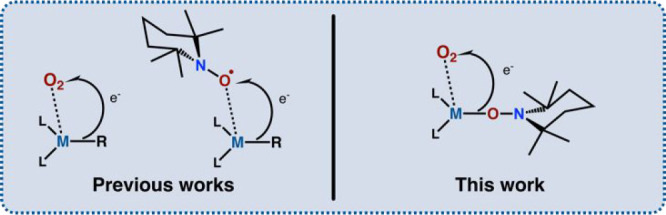
Rationale of the Designed Reaction System for the Activation of O_2_

The activation of O_2_ by the model Ca-TEMPO complex tackles another fundamental issue of the dioxygen activation by various nature-driven systems, the understanding of which is essential in designing biomimetic processes aimed at sustainable development. Dioxygen is a fundamental molecule in life processes and a powerful oxidant in various biological systems involving metalloenzymes.^48^ To provide a more in-depth view of the O_2_ activation pathways, numerous transition metal-based synthetic biomimetic model systems have been developed in the last decades.^49−55^ Conversely, reaction systems based on redox-inactive metal complexes have been paid much less attention and have only started to gain momentum.^56−61^ For example, structural tracking of the O_2_ activation by main group organometallics and their organozinc relatives has emphasized the essential role of the Lewis acidity and coordination state of the oxygenated organometallic compounds.^62,63^ Moreover, systematic studies on the oxygenation of organometallics with redox inactive metal centers laid a foundation for a novel mechanism of the oxygenation process, i.e., the inner sphere electron transfer (ISET) mechanism, which strongly contradicted the textbook radical-chain mechanism.^57,64,65^ The ISET mechanism assumed that a critical step of the oxygenation reaction involves a single electron transfer (SET) from the M-C bond to the noncovalently activated O_2_ molecule (Scheme 2, left). Notably, with regard to the textbook radical chain mechanism, the radical character of the oxygenation process has usually been justified by the inhibition of the oxygenation reaction in the presence of nitroxyl radicals.^66^ However, recent studies demonstrated that the stable nitroxyl radicals smoothly react with organometallics and initiate the liberation of alkyl radicals.^67^ More importantly, reactions of nitroxyl radicals with organometallics likely involve a SET from the M–C bond to the coordinated nitroxyl radical and hence nicely resembles a key step in the novel ISET mechanism (Scheme 2, right).^67,68^

Based on our vast expertise in the oxygenation of organometallics with redox-inactive metal centers^56−58,65,64^ and the multifaced chemistry of organometallic/TEMPO systems,^67,69,70^ we envisioned that the marriage of these two fertile landscapes might be a new vista for the O_2_ activation (Scheme 2). As proof of concept, we present the preparation and structural characterization of the first Ca complex incorporating a TEMPO anion and reveal its reactivity toward O_2_, leading to unique Ca-hydroperoxide and heterometallic Ca/K peroxide complexes. We also propose a mechanistic rationalization for the reaction outcomes based on combined experimental and theoretical investigations. Our synthetic approach demonstrates the simplicity and elegance of metal-TEMPO complex application in the O_2_ activation.

## Results and Discussion

### Synthesis of the First Ca-TEMPO Complex

**Scheme 3:**
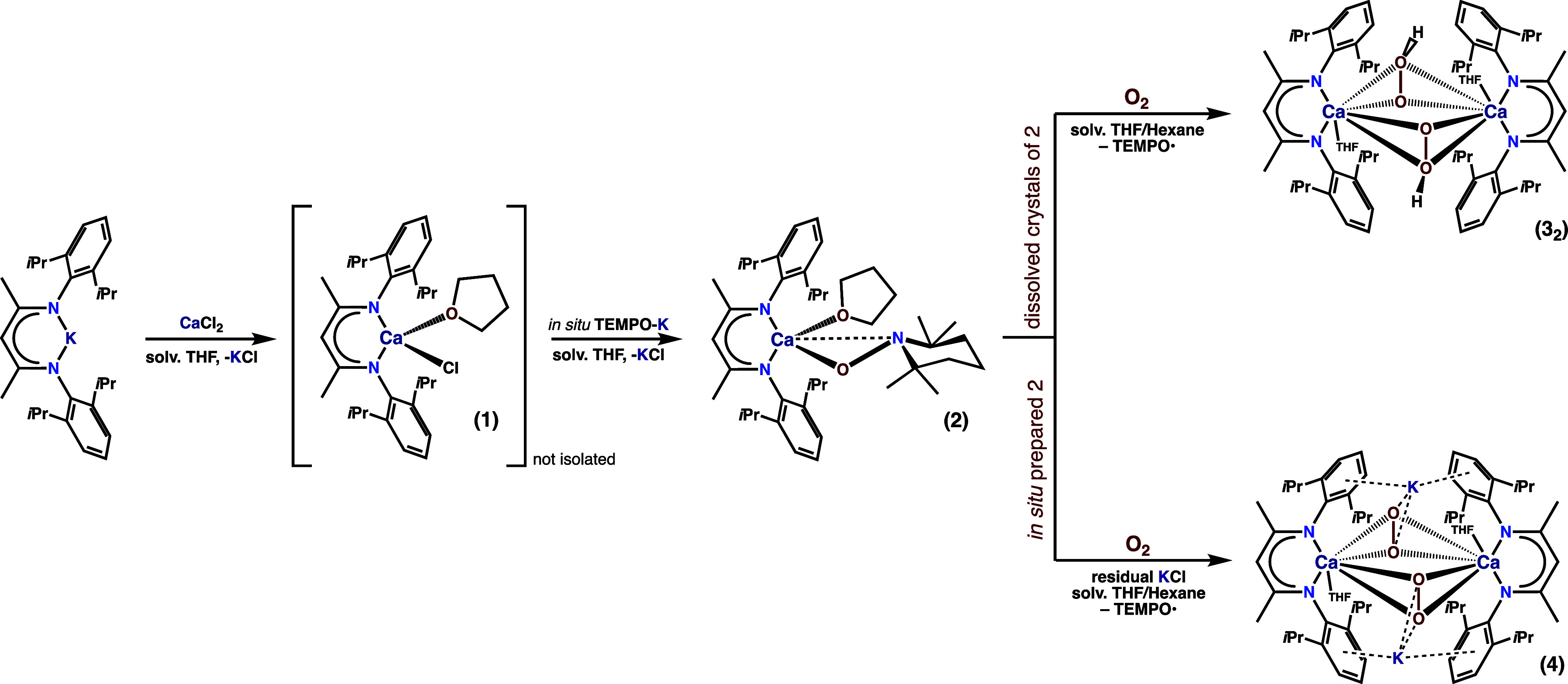
Synthesis of Compounds 1–4

**Figure 1:**
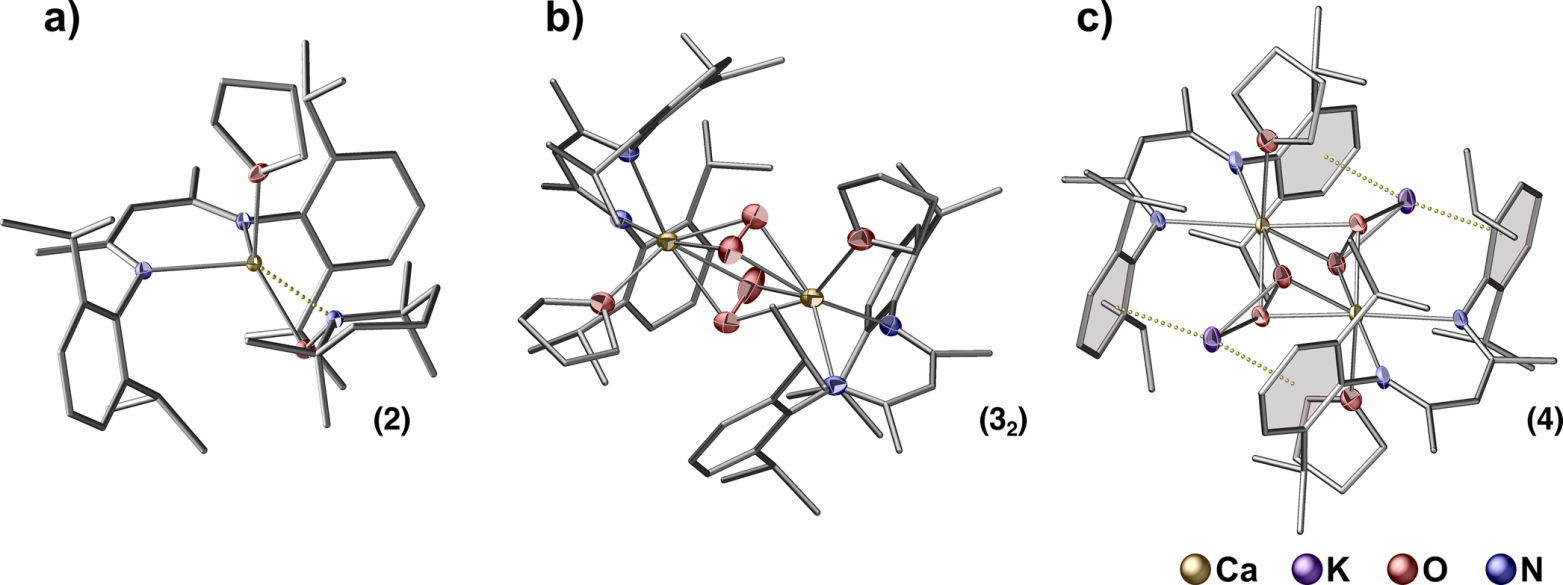
Molecular structures of β-diketiminate supported Ca-TEMPO complex **2** (a), Ca hydroperoxide **3**_**2**_ (b), and Ca peroxide **4** (c). Hydrogen atoms were omitted for clarity.

We selected the ubiquitous β-diketiminate ligand framework as a supporting scaffold due to its common successful use in the stabilization of various long-sought intermediates and highly reactive species,^71−73^ including a vital role in the isolation of elusive calcium complexes.^74−84^ Initially, a Ca chloride precursor [(^dipp^BDI)CaCl(THF)]_*n*_ (**1**) was prepared in situ by reacting equimolar amounts of CaCl_2_ with a K salt of a β-diketiminate ligand (HC{C(Me)N[C_6_H_3_*i*Pr_2_-2,6]}_2_, abbreviated herein as ^dipp^BDI), in tetrahydrofuran (THF), according to the standard protocol. Next, the addition of a (TEMPO)K salt to the in situ prepared solution of **1** followed by the crystallization at 0 °C afforded the targeted complex [(^dipp^BDI)CaTEMPO(THF)] (**2**) with essentially quantitative yield (Scheme 3). Compound **2** was fully characterized spectroscopically (Figures S1, S4–S6) and using single-crystal X-ray diffraction (Figure 1a). In the solid state, **2** exists as a monomer with a severely distorted tetrahedral geometry of a Ca center. The anionic TEMPO ligand coordinates the Ca center in a κ^2^(O,N)-mode, in contrast to the Mg analogue comprising the κ^1^(O)-coordinated TEMPO ligand.^85^ The Ca coordination environment is completed by a THF molecule.

### Synthesis of Ca Peroxide Complexes

Even though metal-TEMPO complexes have been utilized for different homogeneous oxidation reactions, owing to their unique capabilities for electron exchange,^86,87^ their controlled reversible reduction in the presence of O_2_ appears to be an unexplored area of research. We recall at this point that the ISET mechanism for the oxygenation of redox-inactive metal alkyls assumes that a SET from the M-C bond to an O_2_ molecule is crucial in this process.^57^ Given that the TEMPO anion readily undergoes 1e̅ oxidation, we wondered if the Ca-TEMPO complex **2** could act as an efficient system for the reductive activation of O_2_. We hypothesized that the SET from the Ca–O_TEMPO_ bond to O_2_ with the concomitant evolution of a free TEMPO radical might be a source of Ca-supported reactive oxygen species (ROS) (Scheme 2). Thus, we investigated the reactivity of **2** toward O_2_. Slow diffusion of predried air in a hexane/THF solution of crystals of **2** for ca. 2 h at 4 °C led to a gradual reddish coloration of the solution, associated with the activation of O_2_ and the liberation of free TEMPO.^85^ Crystallization from the concentrated reaction mixture afforded a novel hydroperoxide [(^dipp^BDI)Ca(μ-OOH)(THF)]_2_ (**3**_**2**_) with moderate yield (Scheme 3); strikingly, the analogous reaction in a hexane/*d*_8_-THF solution led to a complicated reaction mixture, from which we were not able to isolate or spectroscopically identify the desired product (for a more detailed discussion, vide infra).

Conversely, a dramatically different outcome was observed during the oxygenation of in situ prepared complex **2** in THF. In that case, exposition of the mother liquor to dry air in a hexane/THF mixture for ca. 2 h followed by crystallization at −20 °C yielded a heterometallic Ca/K peroxide [(^dipp^BDI)Ca(μ-OO)K(THF)]_2_ (**4**) with a moderate yield (Scheme 3). We would like to emphasize that calcium complexes with alkali metal countercation-specific stability are known;^88−91^ however, the presence of the potassium ion in **4** appears to be surprising at first glance. To this end, we performed an additional ICP-OES analysis of the filtered mother liquor of **2**, revealing a Ca:K molar ratio of 2:1 (for more information, see Table S3). Thus, the presence of potassium cations in **4** might be reasonably explained by forming a contact ion pair between **2** and KCl in a solution; the respective ^1^H DOSY experiment was less sensitive and did not explain the nature of the postreaction mixture (Figure S7).

### Structure Characterization

Compounds **3**_**2**_ and **4** were fully characterized spectroscopically (Figures S2–S6), and their identities were confirmed by the single-crystal X-ray diffraction. Compound **3**_**2**_ exists as a dimer with bridging hydroperoxide units between the Ca centers, which are flanked by ^dipp^BDI ligands and solvated by THF molecules (Figure 1b). There is a spread of Ca–O bond distances ranging from 2.342 to 2.383 Å (Table S6), and the O–O distances [1.340(4) and 1.348(5) Å] are within the typical range of distances recorded for hydroperoxide moieties and shorter than those observed for well-defined main group metal alkylperoxides.^58,92^ Compound **4** is a centrosymmetric dimer possessing two heptacoordinated Ca centers bridged by the peroxide moieties (Figure 1c). Both units in **4** are also seamed by two K^+^ cations interacting with the peroxide oxygen atoms and the flanked aromatic rings. The Ca–O bond distances fall in a narrow range (2.315–2.328 Å, Table S7), and the O–O bond distance (1.550(3) Å) is longer than that found in compound **3**_**2**_, and the observed differences in the O–O bond lengths likely result from the presence of a potassium ion in **4**. DFT frequency calculations were also conducted to simulate the Raman spectra, thus allowing the O–O stretching vibrations of **3**_**2**_ and **4** at 825 and 790 cm^–1^, respectively, to be assigned (Figure S6). In the case of the Raman spectra of **4**, the distinct signal likely corresponds to the vibrations of ^dipp^BDI ligands coordinated to K^+^ ions can also be distinguished (Figure S5). Moreover, the ^1^H NMR signal at 0.35 ppm attributable to the OO-H proton in **3**_**2**_ was visible (Figure S2).

We note that well-defined metal peroxides obtained from the controlled oxygenation of redox-inactive metal complexes are rather scarce, contrasting to the numerous reports on the isolation of transition metal peroxides. Up to now, only homometallic Mg,^93^ Zn,^94,95^ and Ga^96^ as well as heterometallic {[(Me_3_Si)_2_N]_4_M_2_Mg_2_(O_2_)} (M = K or Li)^97^ peroxides have been described. Undoubtedly, the isolation and structural authentication of the hitherto elusive homo- and heterometallic Ca peroxides fills the gap in the chemistry of main group metal peroxides and also provides the first model system for tracking ROS transformations in the presence of Ca ions. Moreover, unveiling the complexity of the interaction between Ca ions and ROS can contribute to a better understanding of the role of the Ca^2+^ site in the OEC, which remains one of the greatest enigmas within the protein-bound heterometallic Mn_4_CaO_5_ cluster in PSII.^7,29−31^

### Mechanistic Considerations Supported by Quantum Chemical Calculations

**Scheme 4:**
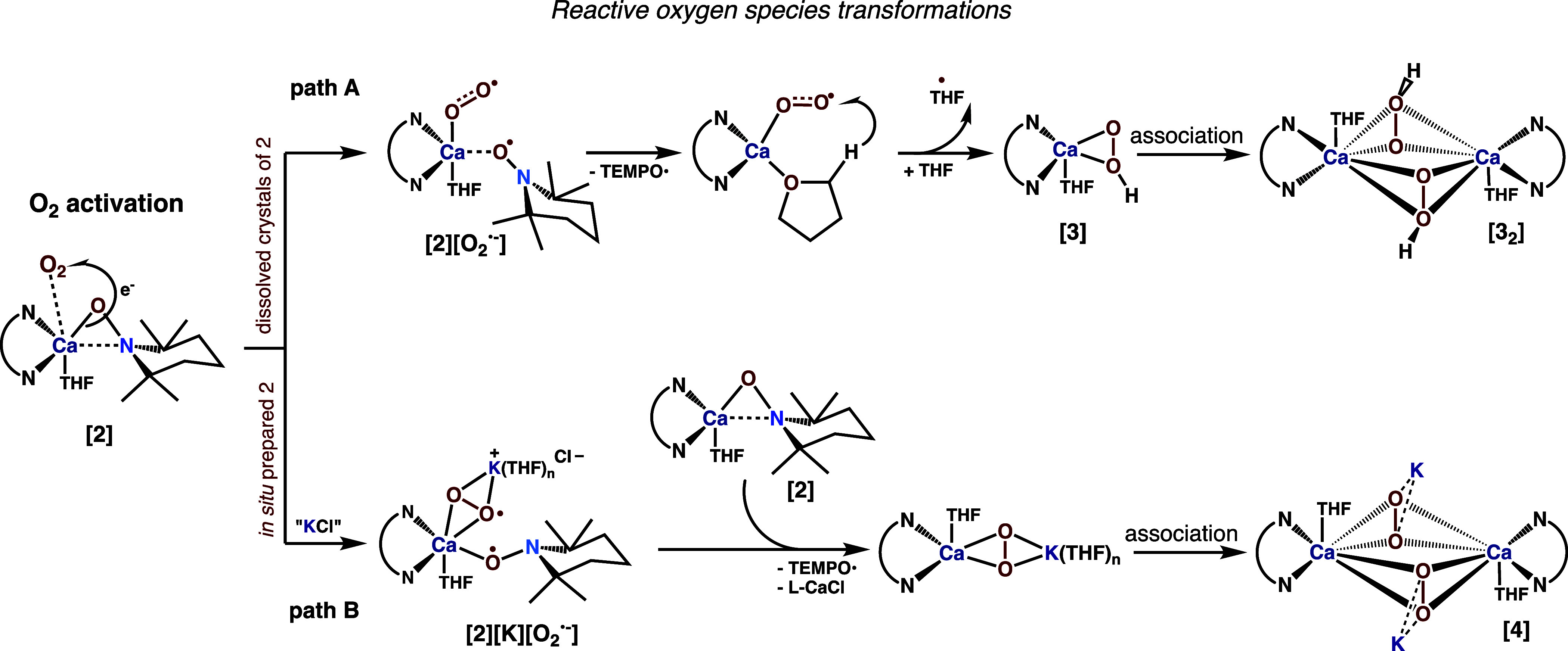
Proposed Reaction Pathways of the Oxygenation of 2 Leading to 3_2_ (*path A*) and 4 (*path B*)

The observed diversity in the reaction outcomes provides a vivid indication that the presence of K^+^ cations appears to be a key factor controlling the oxygenation chemistry of the Ca-TEMPO system. To understand the impact of the synthetic conditions on the transformations involving complex **2**, we proposed the reaction pathways for the oxygenation of **2** as outlined in Scheme 4. In the case of the oxygenation of a pure solution of **2**, the process is initiated by an attack of O_2_ on the Ca center followed by the ET from the Ca–O_TEMPO_ bond to the O_2_ molecule. This stage nicely resembles the ISET mechanism (vide supra) as the HOMO orbital of **2** has a π*(O–N) character with a substantial contribution at the Ca atom (for details, see Table S8). As a result, a highly reactive Ca superoxide [(^dipp^BDI)Ca(OO)(THF)] species is formed, which subsequently abstracts the hydrogen atom from a THF molecule. The resulting hydroperoxide [(^dipp^BDI)Ca(OOH)(THF)] (**3**) moiety is stabilized by the formation of a dimer **3**_**2**_. The proposed hydrogen atom transfer (HAT) from the coordinated THF molecule may not be an obvious reaction pathway, but the observed retardation of this process in the presence of *d*_8_-THF (for dramatic kinetic isotope effect in the HAT process from THF and *d*_8_-THF, see Tolman et al.)^98^ with likely simultaneous shifting to side reactions along with our computational analysis (vide infra) supports this view. Moreover, in recent years, there has been a lively discussion on the HAT processes mediated by superoxide complexes with redox-active metal centers, and the number of well-documented examples is constantly growing.^99−105^

For the Ca-TEMPO/K system, the presence of K^+^ ions in the reaction systems dramatically affects the reaction pathway. In this case, the first step of the oxygenation likely also involves the SET from the Ca–O_TEMPO_ bond to the attacking O_2_ molecule with the formation of a Ca superoxide additionally stabilized by a K^+^ ion (Scheme 4, path B). The ion pair formation likely supports a SET from the second molecule of **2**, forming a monomeric Ca peroxide and liberating free TEMPO. Finally, the subsequent association of the heterometallic peroxides leads to the formation of **4**. Our suspicions of the hydrogen abstraction from THF were supported by GC–MS analysis of postreaction liquors. In the case of the oxidation of dissolved crystals of **2** in THF (cf. Scheme 4, path A), we found 2-hydroxytetrahydrofuran as a reaction product. In turn, the similar GC–MS measurements after the oxygenation of precrystallized THF solution of **2** (cf. Scheme 4, path B) do not reveal THF oxygenation products. We also kept in mind the well-documented noninnocence of β-diketiminate ligands,^73,106^ but the GC-MS experiments do not clearly indicate oxidation products of β-diketiminate units.

To substantiate the proposed mechanistic pathways and provide a rationalization of the experimental observation, we carried out a set of quantum chemical calculations. Geometry optimizations and frequency calculations were carried out within the density functional theory (DFT) using BP86 functional^107^ augmented with the D3BJ dispersion correction^108^ using the def2-SVP basis set^109^ (TURBOMOLE 7.3).^110^ These calculations provided us with the zero-point energy (ZPE) correction. Single point energies were recomputed with the r^2^SCAN-3c composite method of Grimme et al.,^111^ along with the SMD implicit solvation model^112^ (THF) as implemented in the ORCA 5.0 program.^113^ Free K^+^ ions are represented as [K(THF)_4_]^+^, and coordination to any species considered is accompanied by a loss of one THF molecule ([K(THF)_4_]^+^ denoted as **[K**^**+**^**]**). Final reported energies are inclusive of ZPE correction.

**Figure 2:**
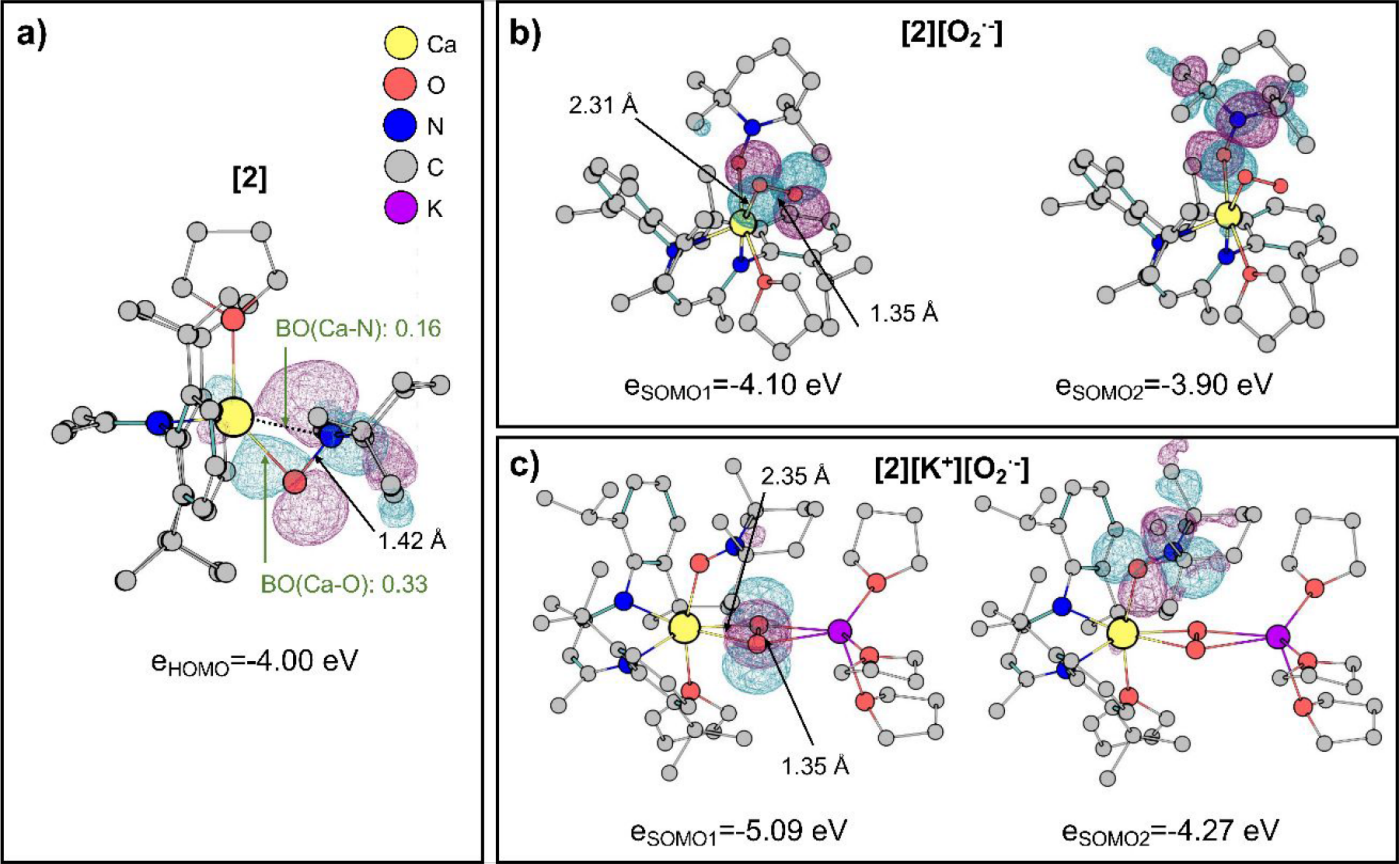
DFT-optimized structures of **2** (a). DFT-optimized structures of the products of reaction with molecular oxygen in the absence of K^+^ ions **[2][O2**^**·–**^**]** (b), and with K^+^ ions **[2][K**^**+**^**][O2**^**·–**^**]** (c), with the key interatomic distances marked in black. In panel (a), the selected Mayer bond orders are highlighted in green. For each structure, the isosurfaces (±0.03 au) of the selected frontier molecular orbitals are drawn along with associated energy (HOMO for closed-shell for **2** and QRO SOMOs for other two molecules in the triplet ground state).

In the parent complex **2**, both the O and N atoms of the coordinated TEMPO display some sort of bonding toward the Ca center with Mayer’s bond order for the Ca–O and Ca–N bonds being 0.33 and 0.16, respectively (Figure 2a). The highest occupied molecular orbital (HOMO) is shared between the O and N atoms and has a π* character. The calculations also support the proposed reaction pathways of the oxygenation of **2**. In the case of the reaction system without K^+^ ions, an interaction of O_2_ with the Ca center is accompanied by the ET from the HOMO orbital of **2** to O_2_ and followed by the formation of a superoxide transient species **[2][O**_**2**_^**·–**^**]** (Scheme 4, path A). The formation of **[2][O**_**2**_^**·–**^**]** is exothermic (−13.4 kcal/mol; reaction energies are listed in Table 1), and this transient complex features the end-on bound O_2_^·^ moiety with the O–O bond length of 1.35 Å (Figure 2b). The results showed that the THF molecule in **[2][O**_**2**_^**·–**^**]** is coordinated in such a way that an H atom transfer to the superoxide moiety is greatly facilitated by a proximity effect and spatial orientation of the respective molecular orbitals (see Figure S12); here, the H atom of the THF is in the plane of the SOMO orbital of O_2^·^_ so that the proximity effect is accounted for in the structure. Thus, complex **[2][O**_**2**_^**·–**^**]** abstracts a H atom from a THF molecule, which leads to a putative Ca hydroperoxide **3** (Scheme 4, path A); we note that the radical-mediated selective C–H functionalization of THF is a well-established process.^68,114^ In turn, the presence of K^+^ ions in the title reaction system significantly influences the oxygenation of **2**. Based on the experimental observation, one may expect that K^+^ ions additionally stabilize the superoxide moiety. Indeed, the O_2_ binding in such case is more exothermic by about 18 kcal/mol (−24.3 kcal/mol for the formation of **[2][K**^**+**^**][O**_**2**_^**·–**^**]**) as compared to the system without K^+^ ions (see Figure 2b,c). Hypothetically, directed radical H-abstraction from the coordinated THF ligand for **[2][K**^**+**^**][O**_**2**_^**·–**^**]** with the formation of hydroperoxide is also feasible. The reaction barrier in this system is found to be 24.8 kcal/mol and is slightly reduced to that calculated for **[2][O**_**2**_^**·–**^**]** (27.9 kcal/mol). However, gripped by the observed reaction outcomes, we wondered what was the driving force behind the divergent oxygenation pathways of **2**. Remarkably, we found that the presence of K^+^ ions significantly decreases the energy of the singly occupied molecular orbitals (SOMOs, see Figure 2b,c). Consequently, we anticipate that the **[2][K**^**+**^**][O**_**2**_^**·–**^**]** complex (*e*_SOMO1_ = −5.09 eV) will be a better electron acceptor than **[2][O**_**2**_^**·–**^**]** (*e*_SOMO1_ = −4.10 eV). The anticipated donor orbital for both reaction systems, HOMO of **2**, is well aligned energetically (*e*_HOMO_ = −4.00 eV, see Figure 2a) so that the expected driving force for SET to **[2][K**^**+**^**][O**_**2**_^**·–**^**]** is larger than for **[2][O**_**2**_^**·–**^**]**.

**Table 1:** Reaction Energies in kcal/mol Computed at the r^2^SCAN-3c Level with SMD Solvation Model (THF) and ZPE Correction

reaction	Δ*E*
**2** + O_2_ → **[2][O**_**2**_^**·–**^**]**	–13.4
**[2][K**^**+**^**]** + O_2_ → **[2][K**^**+**^**][O**_**2**_^**·–**^**]**	–24.3
**2** + **2** → **[2]**_**2**_	–5.6
**[2]**_**2**_ + KCl(THF)_4_ → **[2]**_**2**_**[KCl]** + THF	–8.0

To further check if the ET process is favored in the case of **[2][K**^**+**^**][O**_**2**_^**·–**^**]** (consistent with Scheme 4, path B), we considered weakly interacting paramagnetic dimeric species **[2a][O**_**2**_^**·–**^**][2]** and **[2a][O**_**2**_^**·–**^**][KCl][2]**, where **[2a]** is used to mark the moiety where free TEMPO was removed. In both systems, the SET process can occur from **[2]** to **[O**_**2**_^**·–**^**]**. To investigate the nature of the excited states in these systems with presumably charge transfer (CT) nature, we carried out high-level multireference calculations based on the complete active space self-consistent field (CASSCF)^115^ method augmented with n-electron valence state perturbation theory treatment of the dynamic correlation (NEVPT2).^116^ We found that the S_1_excited states of **[2a][O**_**2**_^**·–**^**][2]** and **[2a][O**_**2**_^**·–**^**][KCl][2]** have the expected CT character and are located at 4.57 and 3.01 eV, respectively. In the Marcus theory picture, the decreased energy of the first CT state translates almost linearly to the increased driving force of the SET. Therefore, consistently with the one-electron picture (SOMO energies), the presence of KCl enhances the probability of electron transfer for **[2][K**^**+**^**][O**_**2**_^**·–**^**],** as compared to **[2][O**_**2**_^**·–**^**],** and opens an alternative route to the direct H-abstraction. Thus, the observed divergent outcomes of the oxygenation of **2** are dictated by the presence or absence of K^+^ ions in the reaction system.

## Conclusions

In summary, we have recently witnessed a revival of interest in Ca chemistry. However, the activation of O_2_ by Ca complexes has not been the subject of thorough studies. The reported results demonstrate the multifaceted nature of metal-TEMPO complexes, which allows them to act as versatile platforms for O_2_ activation. Harnessing the *β*-diketiminate-supported Ca-TEMPO complex with O_2_, the successful synthesis of both the Ca hydroperoxide and the heterometallic Ca/K peroxide has been shown for the first time. The experimental results in tandem with theoretical studies broaden the state-of-the-art of interactions between redox-inactive metal complexes and O_2_,^57,58^ particularly the interplay of Ca^2+^ ions and ROS. The novel Ca peroxides also bear a resemblance to the postulated S_4_ transition state of the Kok catalytic cycle of water splitting and thus might foster a broader discussion on the role of a Ca^2+^ site in the bimetallic oxygen-evolving complex in PSII and support the design of biomimetic complexes for modeling of oxygen-evolving systems.

## Supporting Information Available

Experimental details, synthesis and characterization information, spectroscopic data, crystal data, structure refinement parameters, and DFT calculations (PDF)
