# Supplementary material for: Fresh Impetus in the Chemistry of Calcium Peroxides
Source: J Am Chem Soc. 2024 Jun 7;146(28):18938–47. doi: 10.1021/jacs.4c00906 (PMC11258691; doi:10.1021/jacs.4c00906)
Supplement: Supplementary file 1 — ja4c00906_si_001.pdf [file ja4c00906_si_001.pdf]

Supporting Information

Fresh Impetus in the Chemistry of Calcium Peroxides

Arkadiusz Kornowicz[1]‡, Tomasz Pietrzak[2]‡, Krzesimir Korona[2]‡, Michał Terlecki[2], Iwona Justyniak[1], Adam Kubas[1]* and Janusz Lewiński[1,2]*

[1]Institute of Physical Chemistry, Polish Academy of Sciences, Kasprzaka 44/52, 01-224 , Warsaw, Poland.

[2] Faculty of Chemistry, Warsaw University of Technology, Noakowskiego 3, 00-664, Warsaw, Poland

*Email: janusz.lewinski@pw.edu.pl; akubas@ichf.edu.pl

‡These authors contributed equally to this work.

Table of Contents

1. Synthesis of compounds 2-4 2

3. ^{1}H NMR spectra of compounds 2-4 4

4. Raman spectra of compounds 2-4. 6

5. DOSY measurements and analysis. 7

6. ICP-OES measurements. 8

7. GC-MS measurements. 9

8. Single-crystal X-ray diffraction. 9

9. Quantum-chemical calculations. 14

10. References. 22

1. Synthesis of compounds 2-4

General experimental methods. Unless otherwise stated, all reactions involving air- and moisture-sensitive organometallic compounds were conducted under argon atmosphere using standard Schlenk techniques and glovebox techniques (MBraun UniLab Plus; < 0.1 ppm O_2, < 0.1 ppm H_2O). All glassware was stored in a 150°C oven overnight before use. All solvents were purified by passage through activated aluminium oxide (MBraun SPS) and stored over 4Å molecular sieves. Deuterated solvents were dried over Na/K, distilled under argon atmosphere prior to use and stored over molecular sieves. The spectra were referenced to residual signals of the deuterated solvents. The proligand, ^{Dipp}BDI-H, was synthesised according to literature procedures[1] and deprotonated to ^{Dipp}BDI-K through equimolar reaction with KHMDS. TEMPO-K was synthesised by a reduction of a radical TEMPO by potassium in hexane. $CaCl_2$ in 99.999% purity was purchased from abcr GmbH. All other reagents used in this study were of commercial grade and obtained from Sigma-Aldrich Co. NMR spectra were acquired on a Varian Mercury 400 MHz or Bruker 300 MHz spectrometer at 298K. FT-IR spectra were acquired on a Bruker TENSOR II FTIR Spectrometer. FTIR spectra Raman spectra were collected on a Nicolet Almega Raman dispersive spectrometer. Elemental analysis were performed using an UNICUBE (Elementar Analysensysteme GmbH). ICP-OES measurements were performed using PerkinElmer Avio 550 Max ICP-OES. GC-MS analysis was performed on an Agilent 6890N chromatograph coupled with an Agilent 5973N MS detector.

Synthesis of [(^{Dipp}BDI)Ca(TEMPO)(THF)] (2). A starting [(^{Dipp}BDI)CaCl]$_n$ (**1**) complex was prepared *in situ* by adding a solution of ^{Dipp}BDI-K (456 mg, 1 mmol) to a suspension of $CaCl_2$ (111 mg, 1 mmol) in THF (10 ml). The mixture was stirred overnight. Then, TEMPO-K (196 mg, 1 mmol) was added to the reaction mixture and stirred overnight. Next, KCl was removed by filtration and THF was distilled in vacuo. The residue was dissolved in a 7 ml of hexane and 0.2 ml of THF. The colourless crystals of the product **2** grew in three days at -30°C in *c.a.* 70% yield. **Elemental analysis** (%) calc. for $C_{42}H_{67}N_3O_2Ca$: C 73.53, H 9.84, N 6.12; found: C 73.20, H 9.75, N 6.17; **^{1}H NMR** (300 MHz, C_6D_6, 25°C), δ: 0.43 (s, 6H, ON-C(C$\underline{H}_3$)$_2$), 1.06 (s, 6H, O-NC(C$\underline{H}_3$)$_2$), 1.08-1.17 (tt b, 2H, CH_2-C$\underline{H}_2$-CH_2-*TEMPO*), 1.25 (d, 12H, CH(C$\underline{H}_3$)$_2$), 1.29-1.35 (b, 4H, (C$\underline{H}_2$)$_2$-CH_2-*TEMPO ring*), 1.35-1.43 (m, 12H CH(C$\underline{H}_3$)$_2$, 4H O(CH_2C$\underline{H}_2$)$_2$), 1.75 (s, 6H, C$\underline{H}_3$-*backbone*), 3.30 (m, 4H, C$\underline{H}$(CH_3)$_2$), 3.75 (b, 4H, O(C$\underline{H}_2$$CH_2$)$_2$), 4.77 (s, 1H, C$\underline{H}$-*backbone*), 7.13-7.18 (m, 12H, C$\underline{H}$-*arom*). **^{13}C NMR** (300 MHz, C_6D_6, 25°C), δ: 17.3 (C$\underline{H}_3$-*TEMPO*), 18.7 (C$\underline{H}_3$-*TEMPO*), 24.2 (C$\underline{H}_3$-*backbone*) 24.6 (CH($\underline{C}H_3$)$_2$), 25.1 (CH($\underline{C}H_3$)$_2$), 28.0 (O($CH_2$$\underline{C}H_2$)$_2$), 33.0 ($\underline{C}$H($CH_3$)$_2$), 39.5 ($\underline{C}$-β-*TEMPO ring*), 58.1 ($\underline{C}$-*α-TEMPO ring*), 68.6 (O($\underline{C}H_2$$CH_2$)$_2$), 93.0 ($\underline{C}$H-*backbone*), 123.5 ($\underline{C}$H-*m-arom*), 123.9($\underline{C}$H-*p-arom*), 141.4 ($\underline{C}$-*o-arom*), 147.8 (N$\underline{C}$-*arom*), 165.1 ($\underline{C}$N-*backbone*). **FTIR** *v*/cm^{-1}: 3056(w), 2864(m), 1738(w), 1544(m), 1513(m), 1461(s), 1430(s), 1404(vs), 1383(s), 1363(s), 1348(m), 1341(m), 1312(vs), 1276(m), 1252(m), 1227(m), 1167(s), 1131(w), 1098(m), 1032(m), 1020(m), 921(m), 880(m), 784(s), 757(s), 742(m), 732(m), 591(m), 483(s), 450(m), 415(m).

Synthesis of [(^{Dipp}BDI)Ca(μ-OOH)(THF)]$_2$ (3_2). The Schlenk vessel with crystals of **2** (137.2 mg, 0.2 mmol) dissolved in hexane/THF (10/0.2 ml) was cooled to -20°C. Then, the vessel was equipped with an open-end adapter with anhydrous $CaCl_2$ and placed in a fridge at 4°C for two hours without stirring. Afterwards, the adapter was changed to a glass stopcock, and the reaction mixture was gently concentrated, slowly poured by pentane (10 ml) and stored at - 30°C. The colourless crystals of **3_2** was collected after a week (yield *c.a.* 30%). **Elemental analysis** (%) calc. for $C_{33}H_{50}CaN_2O_3$: C 70.42, H 8.95, N 4.98; found: C 70.28, H 9.06, N 5.15; **^{1}H NMR** (300 MHz, toluene-d_8, -30°C), δ: 0.30 (s, 1H, $OO\underline{H}$), 1.22-1.39 (m, 24H, $CH(C\underline{H}_3)_2$), 1.39-1.49 (b, 4H, $O(CH_2C\underline{H}_2)_2$), 1.62 (s, 6H, $C\underline{H}_3$-*backbone*), 3.12 (dd, b, 4H, $C\underline{H}(CH_3)_2$), 3.49 (b, 4H, $O(C\underline{H}_2CH_2)_2$), 4.62 (s, 1H, $C\underline{H}$-*backbone*), 7.12 (b, 6H, $C\underline{H}$-*arom*). **^{13}C NMR** (300 MHz, toluene-d_8, -30°C), δ: 23.8-26.3 ($C\underline{H}_3$-*backbone*, 2 x $CH(\underline{C}H_3)_2$, $O(CH_2\underline{C}H_2)_2$), 28.8 ($CH(\underline{C}H_3)_2$), 29.1 ($CH(\underline{C}H_3)_2$), 32.8($\underline{C}H(CH_3)_2$), 69.4 ($O(\underline{C}H_2CH_2)_2$), 93.9 ($\underline{C}H$-*backbone*), 123.6-124.7 ($\underline{C}$-*arom*), 136.7 ($\underline{C}$-*arom*), 141.7 ($\underline{C}$-*arom*), 147.3 ($\underline{C}$-*arom*), 168.3 ($\underline{C}N$-*backbone*). **FTIR** *v*/cm^{-1}: 3054(w), 2867(m), 1738(w), 1541(m), 1511(m), 1456(s), 1429(s), 1402(vs), 1364(s), 1313(vs), 1269(m), 1254(m), 1226(m), 1168(s), 1098(m), 1037(s), 1018(m), 924(m), 877(m), 784(s), 756(s), 744(m), (w), 442(m). The analogous reaction in a hexane/d_8-THF solution led to a complicated reaction mixture, from which we were not able to isolate or spectroscopically identify the desired product.

Synthesis of [(^{Dipp}BDI)Ca(μ-OO)K(THF)]$_2$ (4). The starting complex **2** was prepared *in situ* by adding a solution of ^{Dipp}BDI-K (456 mg, 1 mmol) to a suspension of $CaCl_2$ (111 mg, 1 mmol) in THF (10 ml) in the first step. The mixture was stirred overnight. Next, TEMPO-K (196 mg, 1 mmol) was added to the reaction mixture and stirred overnight. Then, the solution was filtered, and THF was removed by distillation. The residue was dissolved in a 7 ml of hexane and 0.5 ml of THF. The solution was transferred to a Schlenk vessel and cooled to -20°C. Then, the vessel was equipped with an open-end adapter with anhydrous $CaCl_2$ and placed in a fridge at 4°C for two hours without stirring. Afterwards, the adapter was changed to a glass stopcock, and the reaction mixture was gently concentrated, slowly poured by pentane (10 ml) and stored at - 30°C. The colourless crystals of **3** was collected after a week (yield *c.a.* 33%). **Elemental analysis** (%) calc. for $C_{33}H_{49}N_2O_3CaK$: C 65.96, H 8.22, N 4.66; found: C 66.25, H 8.58, N 4.69; **^{1}H NMR** (300 MHz, toluene-d_8, -30°C), δ: 1.13-1.30 (m, 24H, $CH(C\underline{H}_3)_2$), 1.27-1.39 (b, 4H, $O(CH_2C\underline{H}_2)_2$), 1.63 (s, 6H, $C\underline{H}_3$-*backbone*), 2.86 (m, 2H, $C\underline{H}(CH_3)_2$), 3.19 (m, 2H, $C\underline{H}(CH_3)_2$), 3.50 (b, 4H, $O(C\underline{H}_2CH_2)_2$), 4.62 (s, 1H, $C\underline{H}$-*backbone*), 7.13 (s, 6H, $C\underline{H}$-*arom*). **^{13}C NMR** (300 MHz, toluene-d_8, -30°C), δ: 23.5-26.5 ($C\underline{H}_3$-*backbone*, 2 x $CH(\underline{C}H_3)_2$, $O(CH_2\underline{C}H_2)_2$), 28.8 ($CH(\underline{C}H_3)_2$), 29.1 ($CH(\underline{C}H_3)_2$), 32.8($\underline{C}H(CH_3)_2$), 68.6 ($O(\underline{C}H_2CH_2)_2$), 93.8 ($\underline{C}H$-*backbone*), 123.8 ($\underline{C}$-*arom*), 124.2($\underline{C}$-*arom*), 136.7 ($\underline{C}$-*arom*), 147.2 ($\underline{C}$-*arom*), 165.0 ($N\underline{C}$-*backbone*). **FTIR** *v*/cm^{-1}: 3054(w), 2866(m), 1623(w), 1544(m), 1510(m), 1457(s), 1430(s), 1404(vs), 1382(s), 1362(s), 1313(s), 1271(m), 1254(m), 1226(m), 1166(s), 1099(m), 1037(s), 1018(m), 925(m), 892(w), 784(s), 754(s), 732(m), 721(m), 695(m), 508(w), 437(m).

3. ^{1}H NMR spectra of compounds 2-4

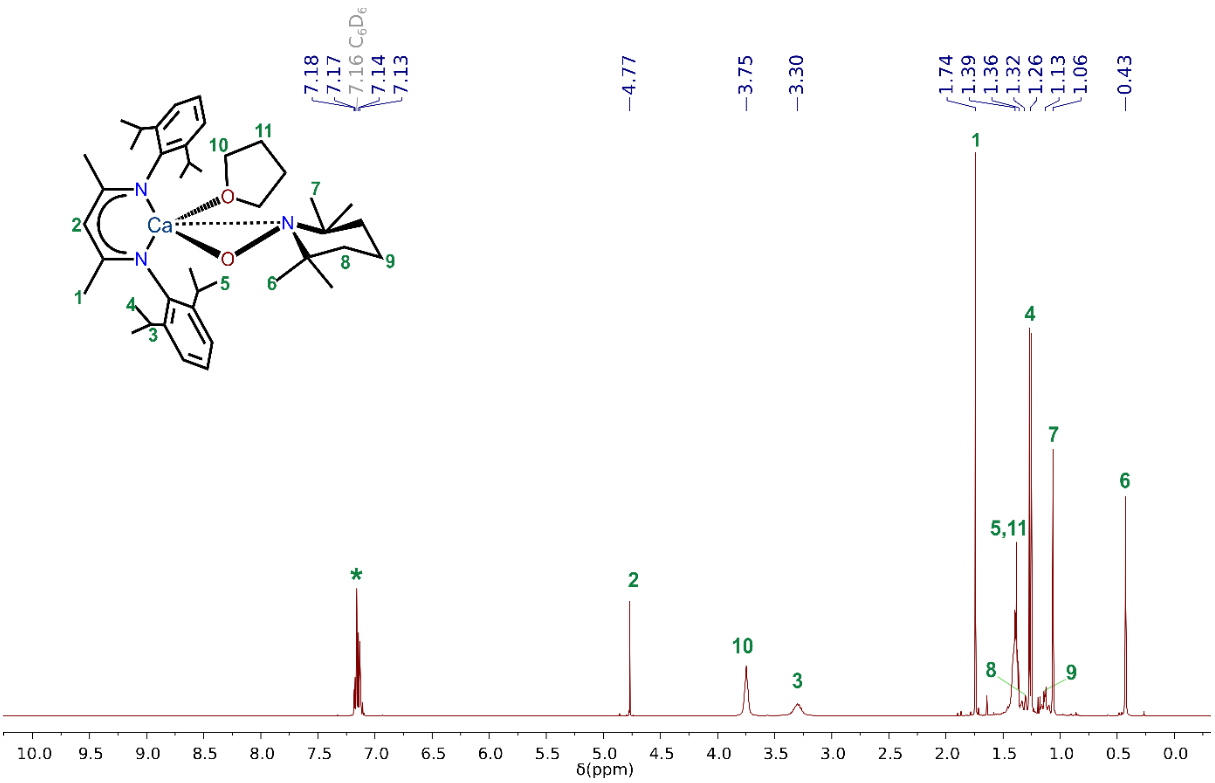

Figure S1. ^{1}H NMR spectrum of **2** in C_6D_6 in rt. The solvent signal is marked by asterisk.

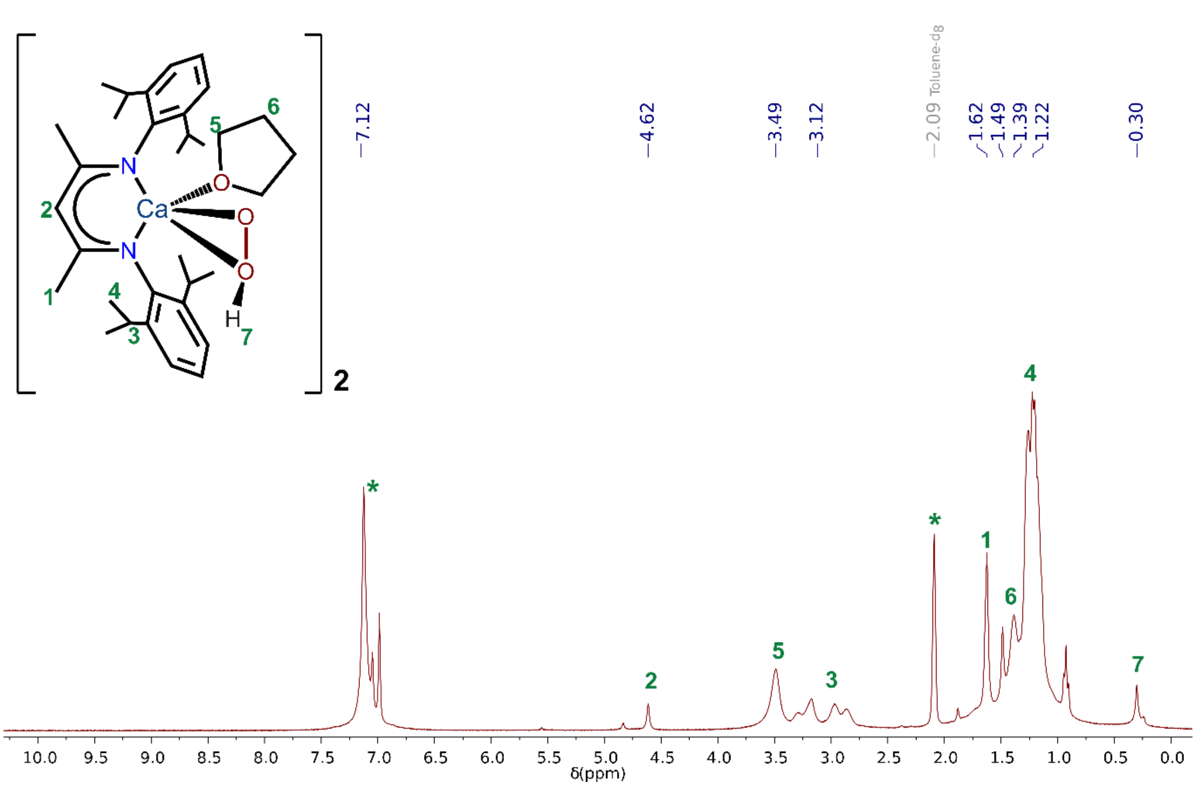

Figure S2. ^{1}H NMR spectrum of $\mathbf{3_2}$ in toluene-d_8 at -30°C.

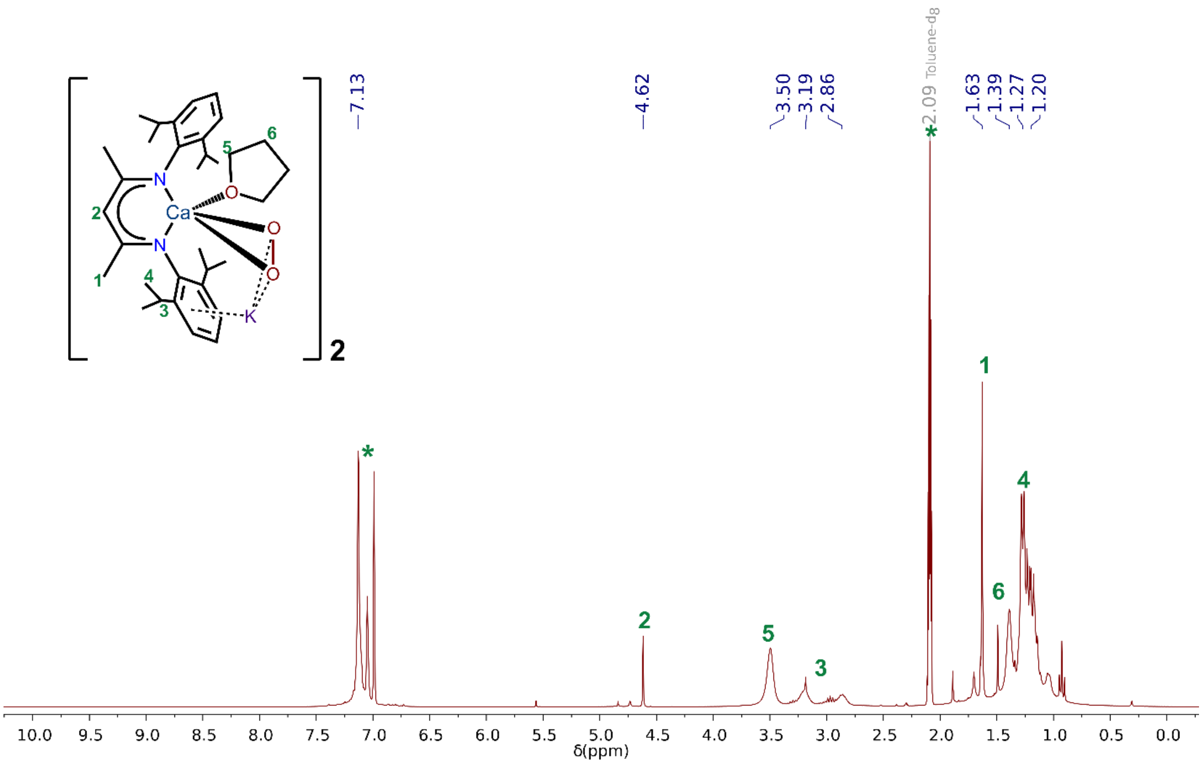

Figure S3. ^{1}H NMR spectrum of **4** in toluene-d$_8$ at -30°C.FTIR spectra of compounds 2-4.

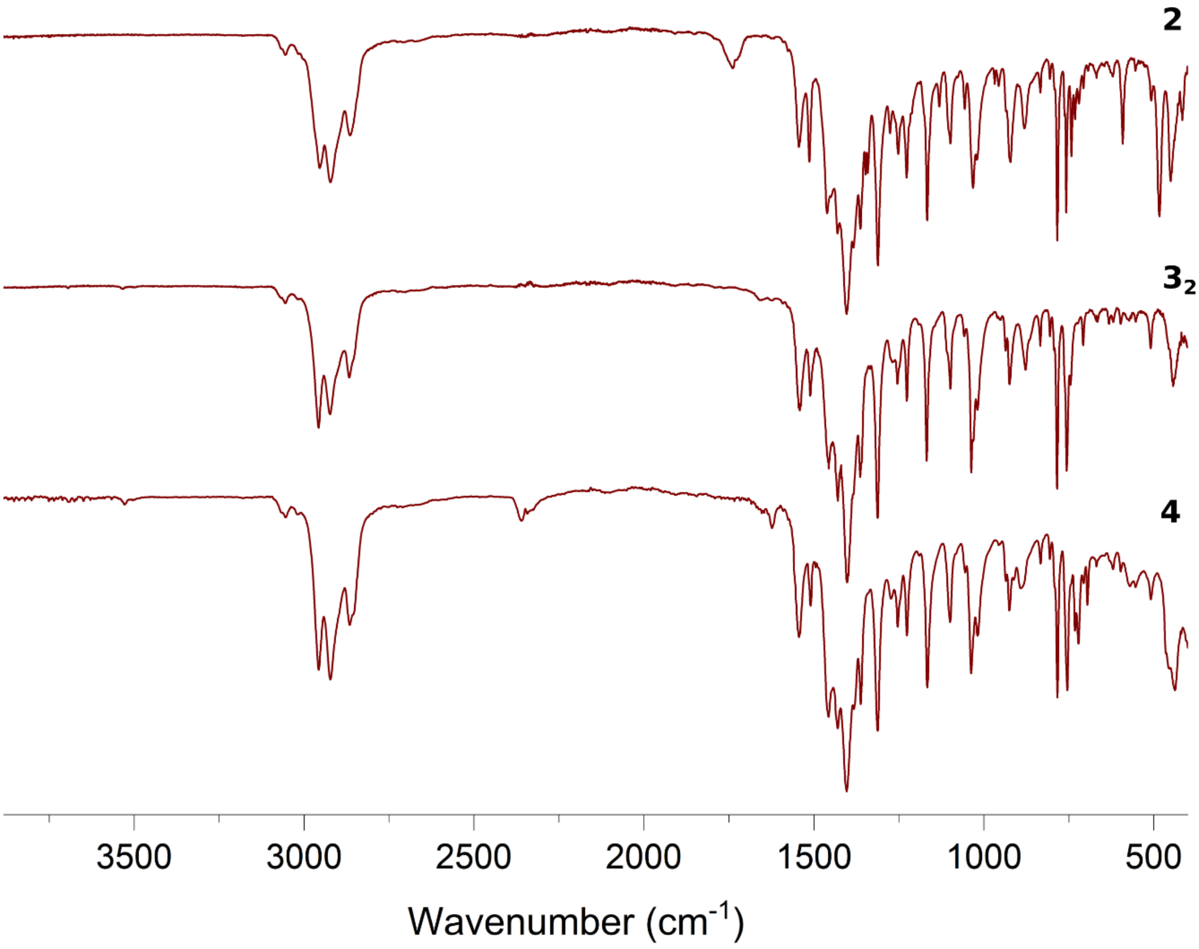

Figure S4. Stacked ATR-FTIR spectrum of **2-4** in nujol.

4. Raman spectra of compounds 2-4.

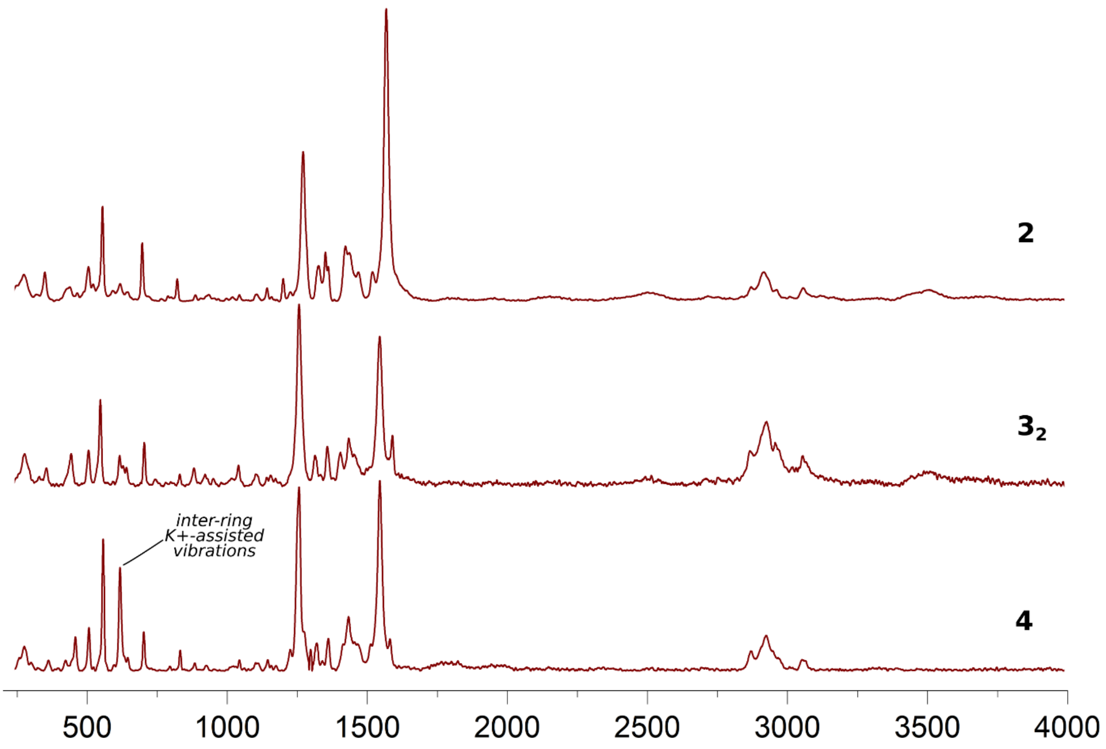

Figure S5. Stacked Raman spectra of **2-4**. The K-assisted inter-ring vibrations characteristic only for compound **4** are marked.

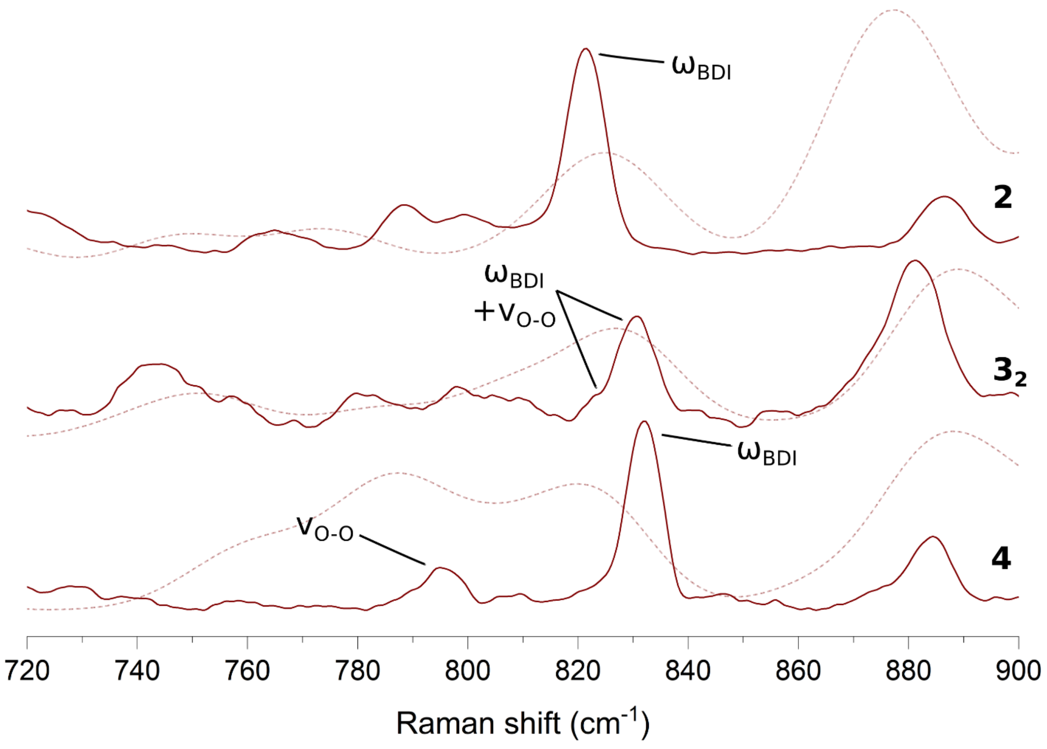

Figure S6. Stacked Raman spectra of **2-4** in a range of 720-900 cm^{-1}. The solid lines correspond to the measured spectra, and the dashed lines illustrate the estimated spectra by DFT calculations. ω_{BDI} correlates to wagging vibrations from N-C_{arom}-C_{arom} bonds. The band from the O-O bond and the neighbouring band from the BDI ligand were marked.

5. DOSY measurements and analysis.

DOSY spectra were acquired on Bruker AVANCE II (300 MHz) spectrometer at -20°C. The molecular weights (*MW*) of analyzed compounds were estimated utilizing an external calibration curve (ECC) approach with normalized diffusion coefficients exploiting 1,2,3,4-tetraphenylnaphtalene (TPhN) as an internal reference.[2] The molecular masses calculated for considered calcium complexes were corrected by a correction factor χ_{cor} for molecules with a high van der Waals density (MDw).[3] The sample for the DOSY experiment was prepared as followed: TEMPO-K was added to a solution of [(BDI)CaCl] in THF. The reaction mixture was stirred overnight and then filtered. The received solution was dried under vacuum. The solid residue was dissolved in THF-d_8 and filtered into the NMR tube.

Analysis of the DOSY spectrum (Figure S7) indicates the presence of a main component of the mixture with an average estimated *MW* of 563 g/mol, which matches well with [(BDI)Ca(TEMPO)] (calculated MW_{cor} = 586 g/mol). However, the high estimated *MW* of 976 g/mol associated with additional resonances at c.a. 3.40 ppm suggests the presence of other larger aggregates, which signals may highly overlap with the spectrum of **2**. Taking into account the significant content of potassium in the solution, as evidenced by ICP-OES (*vide infra*), these aggregates may contain incorporated KCl moieties like e.g. [(BDI)Ca(TEMPO)]•KCl•3THF (MW_{cor} = 846 g/mol) or $[(BDI)Ca(TEMPO)]_2$•KCl (MW_{cor} = 1089 g/mol). The results of calculations are presented in Tables S1-2.

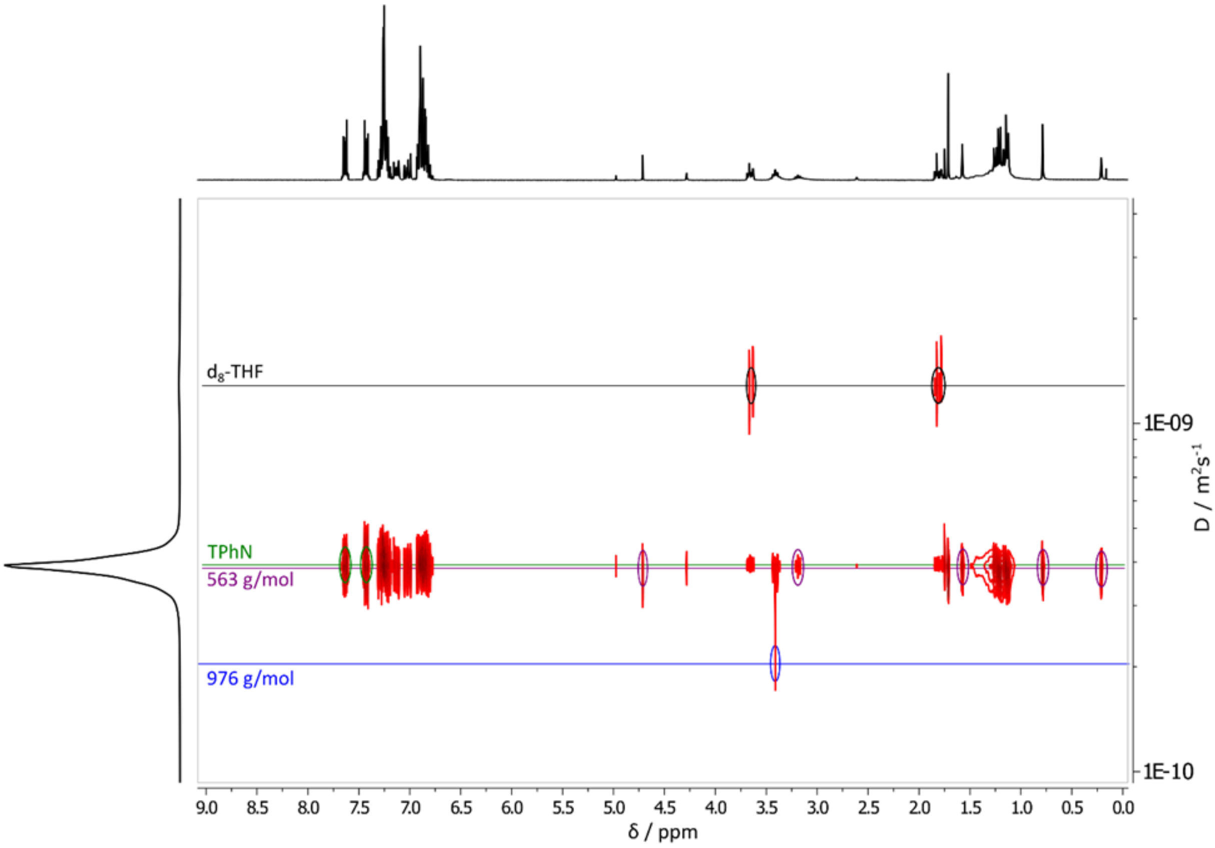

Figure S7. DOSY spectrum of the generated *in-situ* [(BDI)Ca(TEMPO)] in THF-d_8 (signals used to estimate *MW* are marked with asterisks).

Table S1. Calculated molecular weights (*MW*), van-der Waals densities (MD_W), and corrected molecular weights (MW_{cor}) of respective compounds.

Compound	*MW* [g·mol^{-1}]	MD_W [g·mol-1·m^{-3}]	χ_{cor}	MW_{cor} [g·mol^{-1}]
[(BDI)Ca(TEMPO)]	614	4.64·1029	1.05	586
[(BDI)Ca(TEMPO)]•KCl•3THF	905	4.77·1029	1.07	846
$[(BDI)Ca(TEMPO)]_2$•KCl	1303	4.74·1029	1.06	1089

Table S2. Determined diffusion coefficients *D* and estimated molecular weights *MW* based on analysis of DOSY spectrum of the *in-situ* generated [(BDI)Ca(TEMPO)] in d_8-THF.

δ [ppm]	*D* [m^2·s^{-1}]	δ [ppm]	*D* [m^2·s^{-1}]	*MW* [g·mol^{-1}]
Internal reference (TPhN)		[(BDI)Ca(TEMPO)]		
7.60-7.68	$3.92\cdot10^{-10}$	4.69-4.73	$3.55\cdot10^{-10}$	589
7.38-7.47	$3.93\cdot10^{-10}$	3.06-3.26	$3.83\cdot10^{-10}$	516
Average Reference *D*	$3.925\cdot10^{-10}$	1.67-1.73	$3.64\cdot10^{-10}$	564
		0.73-0.83	$3.66\cdot10^{-10}$	559
		0.17-0.26	$3.56\cdot10^{-10}$	586
		Average Estimated *MW*		**563**
		[(BDI)Ca(TEMPO)]•KCl•3THF		
		3.35-3.48	$2.64\cdot10^{-10}$	**976**
		THF		
		3.58-3.72	$1.32\cdot10^{-9}$	62

6. ICP-OES measurements.

To demonstrate the effect of the preparation procedure on potassium contamination in [(BDI)Ca(TEMPO)] reagents we performed ICP-OES measurements for a pure crystalline sample of **2** and of *in-situ* generated [(BDI)Ca(TEMPO)] (Table S3).

The *in-situ* generated sample was prepared as followed: TEMPO-K was added to a solution of **1** in THF. The reaction mixture was stirred overnight and then filtered. The received solution was dried under vacuum. Next, the solid residue was extracted with hexane and dried under vacuum.

The solid samples were finally treated with concentrated HNO_3 and analyzed by PerkinElmer Avio 550 Max ICP-OES.

Table S3. K and Ca concentration in the samples a pure crystalline sample of **2** and the *in-situ* generated [BDICaTEMPO] reagent.

Sample	Concentration [mg/L]	
	K	Ca
crystals of **2**	1.9092	97.2342
in-situ generated [(BDI)Ca(TEMPO)]	42.1057	97.4763

7. GC-MS measurements.

The samples for GC-MS measurements were prepared as follows. The post-reaction liquor was hydrolised by the saturated aqueous solution of $KHCO_3$. The organic phase was separated and dried with $MgSO_4$ overnight. The GC-MS analysis was performed on an Agilent 6890N chromatograph coupled with an Agilent 5973N MS detector.

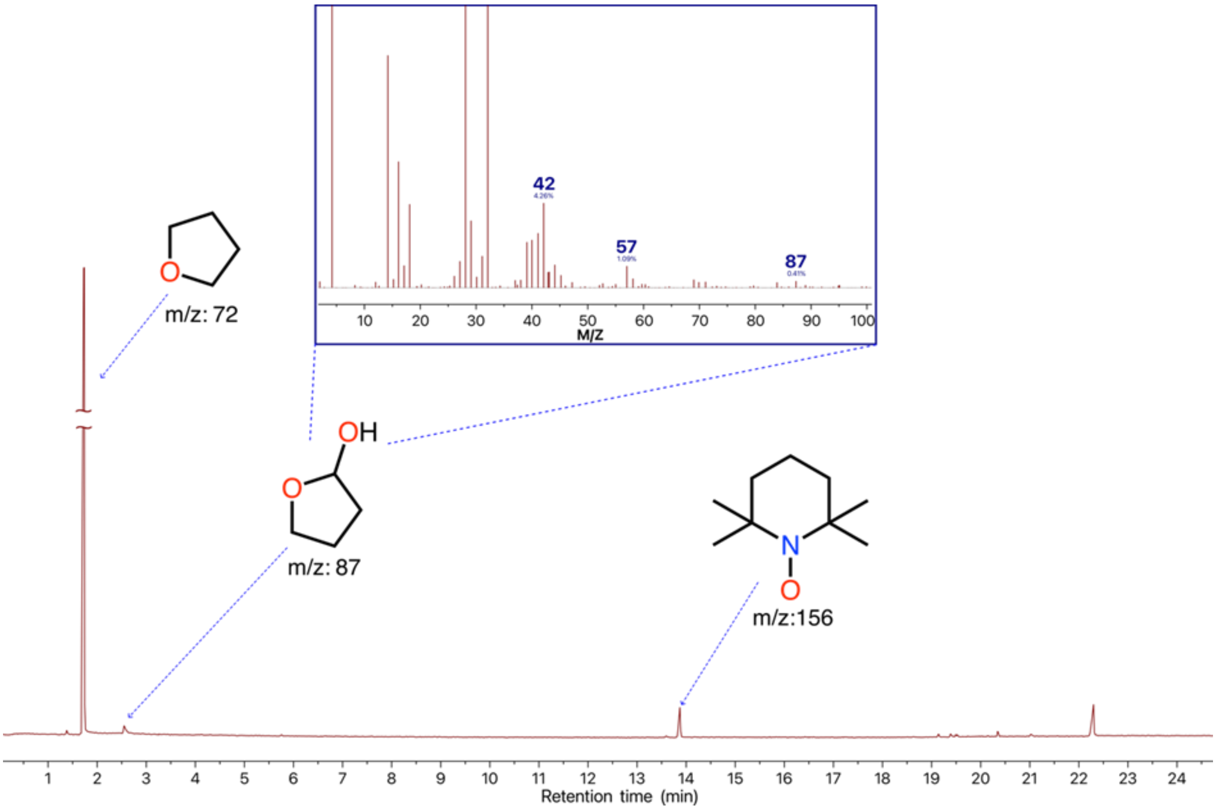

Figure S8. GC-MS analysis of the mother liquor after the oxygenation of dissolved crystals of **2** in THF; we note that the sample preparation method and the measurement specificity allowed only for the qualitatively interpretation of the results.

8. Single-crystal X-ray diffraction.

The crystals of all complexes were selected under Paratone-N oil, mounted on the nylon loops and positioned in the cold stream on the diffractometer. The X-ray data for reported complexes were collected at 100(2)K on a SuperNova Agilent diffractometer and using Mo*Ka* radiation (l = 0.71073 Å) (for compounds **2** and **4**) and using Cu*Ka* radiation (l = 1.54184 Å) (for compound $\mathbf{3_2}$). The data were processed with CrysAlisPro.[4] The structures **2,** $\mathbf{3_2}$ and **4** were solved by direct methods using the SHELXT program and were refined by full matrix least–squares on F^2 using the program SHELXL.[5] All non-hydrogen atoms were refined with anisotropic displacement parameters. Hydrogen atoms were added to the structure model at geometrically idealised coordinates and refined as riding atoms.

In comp **2** and **4** no satisfactory structural models for highly disordered solvent molecules could be assigned, and therefore the Solvent Masking in OLEX2 was used to remove the electron densities of these disordered species.[6]

Crystallographic data (excluding structure factors) for the structure reported in this paper have been deposited with the Cambridge Crystallographic Data Centre as a supplementary publication. Copies of the data can be obtained free of charge on application to CCDC, 12 Union Road, Cambridge CB21EZ, UK (fax: (+44)1223-336-033; e-mail: deposit@ccdc.cam.ac.uk). CCDC: 2286821 (**2**), 2286822 (**3₂**), 2286823 (**4**).

Table S4. Crystallographic data and structure refinement parameters for **2**, **3_2** and **4**.

Identification code	**2**	**3_2**	**4**
Empirical formula	$C_{42}H_{67}CaN_3O_2$	$C_{66}H_{100}Ca_2N_4O_6$	$C_{66}H_{98}Ca_2K_2N_4O_6$
Formula weight	686.06	1125.65	1201.84
Temperature/K	100(2)	100(2)	100(2)
Crystal system	orthorhombic	monoclinic	monoclinic
Space group	*P*bca	*C*2/c	*P*2₁/c
a/Å	16.0888(3)	48.6309(15)	12.1806(8)
b/Å	21.4139(3)	12.4671(4)	14.6463(5)
c/Å	25.7451(4)	22.0061(5)	22.4974(7)
α/°	90	90	90
β/°	90	102.838(3)	92.718(4)
γ/°	90	90	90
Volume/Å^3	8869.8(2)	13008.5(7)	4009.0(3)
Z	8	8	2
ρ_{calc}g/cm^3	1.028	1.150	0.996
μ/mm^{-1}	0.175	1.914	0.288
F(000)	3008.0	4896.0	1296.0
Crystal size/mm^3	0.16 × 0.11 × 0.08	0.11 × 0.07 × 0.03	0.10 × 0.08 × 0.04
Radiation	Mo Kα (λ = 0.71073)	Cu Kα (λ = 1.54184)	MoKα (λ = 0.71073)
Theta range for data collection /°	6.268 to 58.378	8.206 to 133.202	6.642 to 51.998
Index ranges	-21 ≤ h ≤ 21, -23 ≤ k ≤ 28, -34 ≤ l ≤ 29	-57 ≤ h ≤ 57, -14 ≤ k ≤ 14, -24 ≤ l ≤ 26	-15 ≤ h ≤ 14, -16 ≤ k ≤ 18, -24 ≤ l ≤ 27
Reflections collected	31482	25087	19946
Independent reflections	10597 [R_{int} = 0.0486]	11462 [R_{int} = 0.0731]	7839 [R_{int} = 0.0459]
Data/restraints/parameters	10597 / 0 / 447	11462/0/723	7839/0/371
Goodness-of-fit on F^2	1.061	0.989	1.029
Final R indexes [I>=2σ (I)]	R_1 = 0.0485 wR$_2$ = 0.1071	R_1 = 0.0676 wR$_2$ = 0.1618	R_1 = 0.0699 *w*R$_2$ = 0.1774
Final R indexes [all data]	R_1 = 0.0751 wR$_2$ = 0.1157	R_1 = 0.1142 wR$_2$ = 0.1871	R_1 = 0.0916 *w*R$_2$ = 0.1947
Largest diff. peak/hole / e Å^{-3}	0.32/-0.28	0.50/-0.70	1.04/-0.46

$^aR_1 = \Sigma||F_o| - |F_c||/\Sigma|F_o|$. $^b wR_2 = [\Sigma w(F_o^2 - F_c^2)^2/\Sigma w(F_o^2)^2]^{1/2}$, where $w = 1/[\sigma^2(F_o^2) + (aP)^2 + bP]$, $P = (F_o^2 + 2F_c^2)/3$

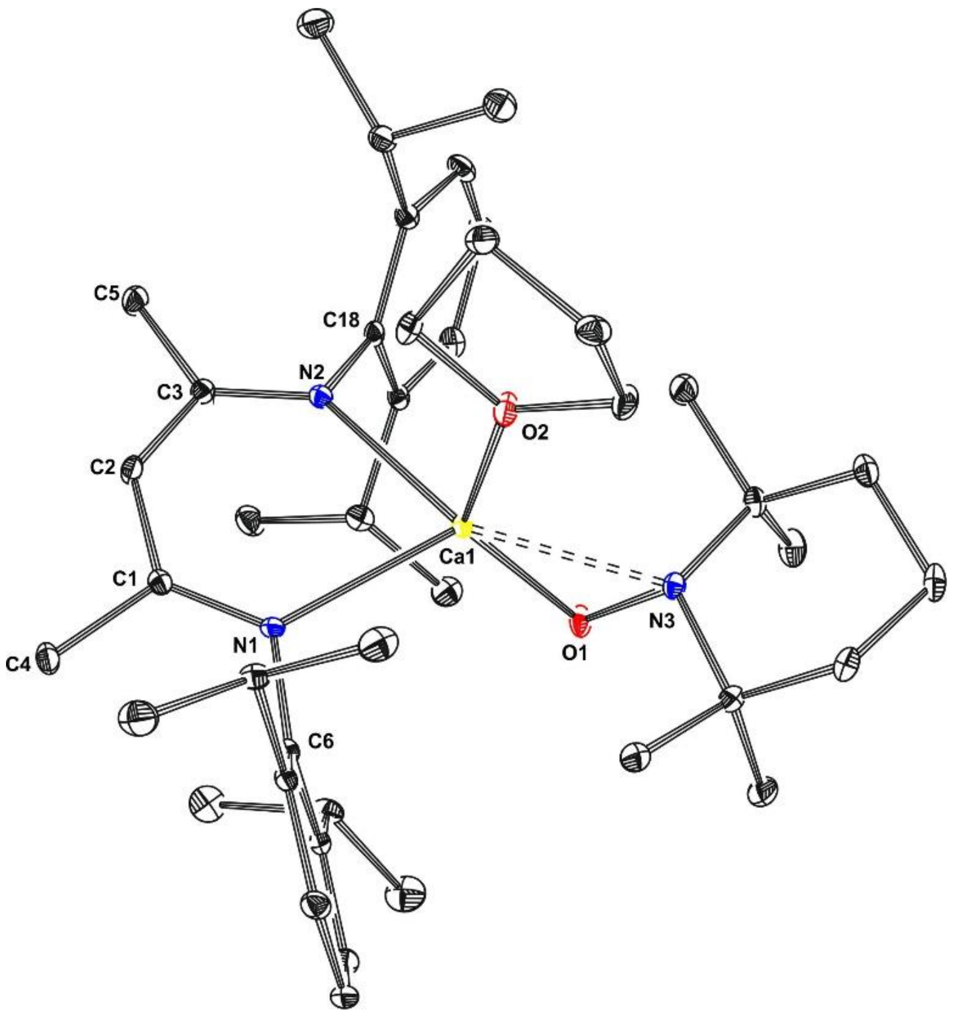

Figure S9. The molecular structure of **2** with thermal ellipsoids set at 30% probability. Hydrogen atoms have been omitted for clarity.

Table S5. Selected intermolecular bond lengths [Å] and angles [deg] for **2**.

**Bond Lengths (Å)**			
Ca1-O1	2.1637(11)	Ca1-N2	2.4071(13)
Ca1-O2	2.3602(11)	Ca1-N3	2.4758(12)
Ca1-N1	2.4083(12)	O1- N3	1.4481(16)
**Bond Angles (deg)**			
Ca1-O1-N3	84.06(7)	N1-Ca1-O1	115.57(4)
O1-Ca1-O2	129.89(4)	N1-Ca1-N3	136.41(4)
O1-Ca1-N1	115.57(4)	N2-Ca1-O2	100.37(4)
O1-Ca1-N2	116.73(4)	N2-Ca1-N3	137.02(4)
N1-Ca1-N2	78.75(4)	O2-Ca1-N3	94.33(4)

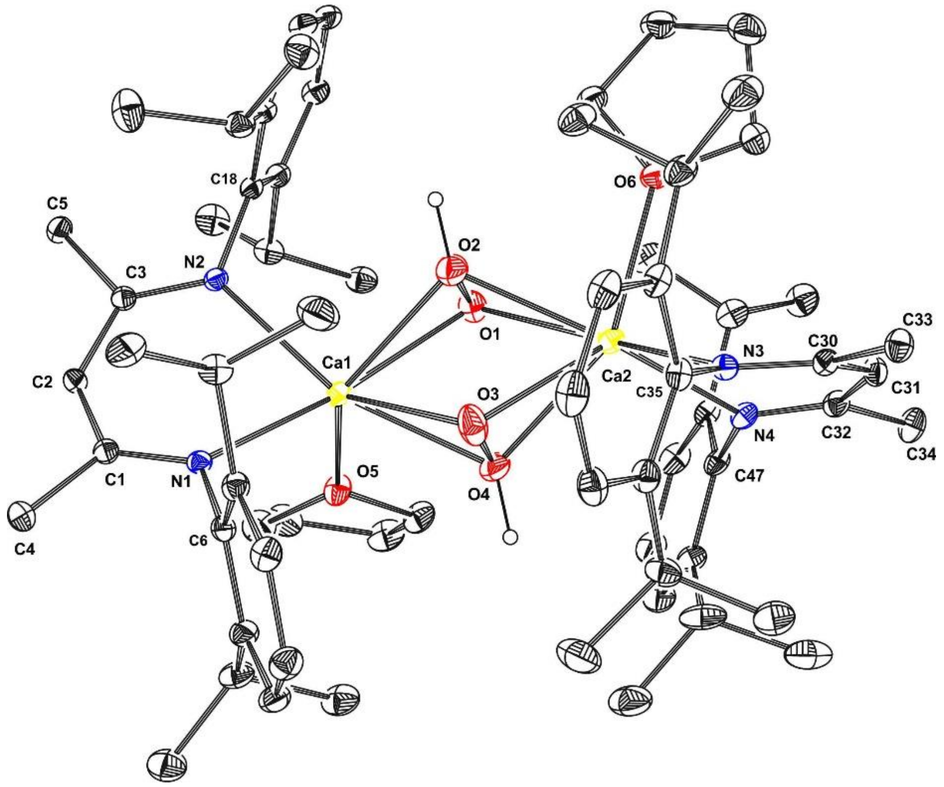

Figure S10. The molecular structure of $\mathbf{3_2}$ with thermal ellipsoids set at 30% probability. Hydrogen atoms have been omitted for clarity.

Table S6. Selected intermolecular bond lengths [Å] and angles [deg] for $\mathbf{3_2}$.

**Bond Lengths (Å)**					
Ca1-O1	2.356(3)	Ca1-N2	2.398(3)	Ca2-N3	2.366(3)
Ca1-O2	2.342(3)	Ca2-O1	2.373(3)	Ca2-N4	2.388(3)
Ca1-O3	2.374(3)	Ca2-O2	2.378(3)	O1-O2	1.340(4)
Ca1-O4	2.382(3)	Ca2-O3	2.369(3)	O3-O4	1.348(5)
Ca1-O5	2.353(2)	Ca2-O4	2.388(3)		
Ca1-N1	2.374(3)	Ca2-O6	2.379(3)		
**Bond Angles (deg)**					
Ca1-O1-Ca2	108.53(12)	O3-Ca1-O5	114.82(12)	O2-Ca2-O6	80.83(10)
Ca1-O2-Ca2	108.84(12)	O4-Ca1-N1	118.19(12)	O3-Ca2-N3	95.59(11)
Ca1-O3-Ca2	108.09(13)	O4-Ca1-N2	161.50(12)	O3-Ca2-N4	125.99(12)
Ca1-O4-Ca2	108.47(12)	O4-Ca1-O5	83.10(10)	O3-Ca2-O6	136.05(11)
O1-Ca1-N1	166.74(10)	O5-Ca1-N1	99.80(10)	O4-Ca2-N3	79.01(10)
O1-Ca1-N2	98.65(10)	O5-Ca1-N2	101.60(10)	O4-Ca2-N4	99.60(11)
O1-Ca1-O5	93.46(10)	N1-Ca1-N2	78.96(10)	O4-Ca2-O6	148.37(11)
O2-Ca1-N1	133.60(11)	O1-Ca2-N3	162.23(10)	O6-Ca2-N3	96.74(10)
O2-Ca1-N2	92.00(10)	O1-Ca2-N4	118.33(10)	O6-Ca2-N4	97.77(11)
O2-Ca1-O5	126.57(11)	O1-Ca2-O6	85.23(10)	N3-Ca2-N4	79.01(10)
O3-Ca1-N1	102.16(11)	O2-Ca2-N3	129.99(11)		
O3-Ca1-N2	142.53(12)	O2-Ca2-N4	151.00(11)		

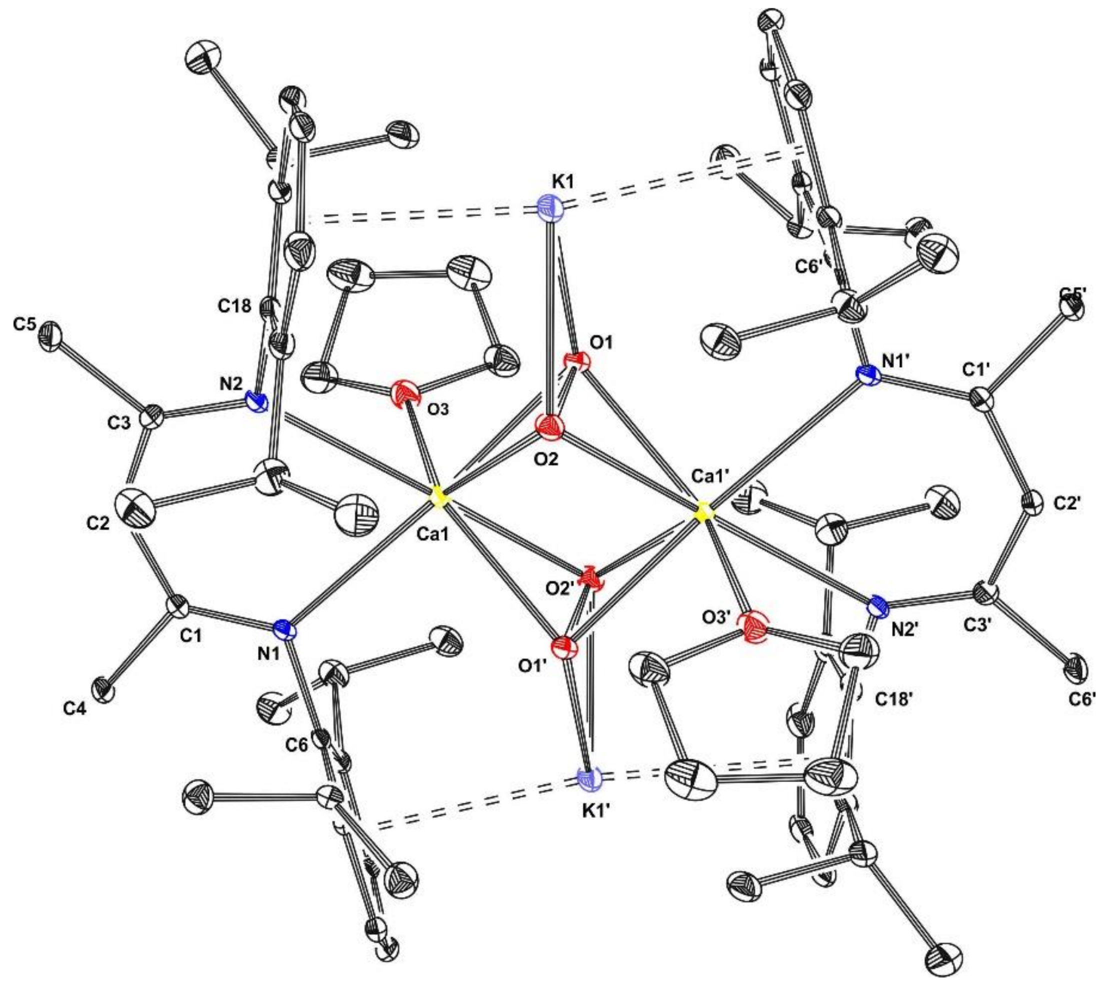

Figure S11. The molecular structure of **4** with thermal ellipsoids set at 30% probability. Hydrogen atoms have been omitted for clarity. Operators for generating equivalent atoms: (*-x+1, -y+1, -z+1*).

Table S7. Selected intermolecular bond lengths [Å] and angles [deg] for **4**.

**Bond Lengths (Å)**			
Ca1-O1	2.315(2)	Ca1-N2	2.531(3)
Ca1-O1’	2.324(2)	O1-K1	2.487(2)
Ca1-O2	2.328(2)	O2-K1	2.497(2)
Ca1- O2’	2.320(2)	O1-O2	1.550(3)
Ca1-N1	2.529(2)		
**Bond Angles (deg)**			
Ca1-O1-Ca1’	86.88(7)	O2-Ca1-N2	92.54(8)
Ca1-O2-Ca1’	86.67(7)	O2-Ca1-O3	124.40(8)
O1-Ca1-N1	175.59(9)	O3-Ca1-N1	88.68(8)
O1-Ca1-N2	102.15(8)	O3-Ca1-N2	85.47(9)
O1-Ca1-O3	87.20(8)	Ca1-O1-K1	120.64(9)
O2-Ca1-N1	144.42(8)	Ca1-O2-K1	119.67(10)
N1-Ca1-N2	75.93(8)		

9. Quantum-chemical calculations.

Geometry optimizations and frequency calculations were carried out within the density functional theory (DFT) using BP86 functional[7] augmented with the D3BJ dispersion correction[8] using def2-SVP basis set[9] (TURBOMOLE 7.3)[10]. These calculations provided us with the zero-point energy (ZPE) correction. Single point energies were recomputed with the r^2SCAN-3c[11] composite method of Grimme et al. along with SMD implicit solvation model[12] (THF) as implemented in the ORCA 5.0 program[13]. Free K+ ions are represented as $[K(THF)_4]^+$, and coordination to any species considered is accompanied by a loss of one THF molecule ($[K(THF)_4]^+$ denoted as **$[K^+]$**). Final reported energies are inclusive of ZPE correction. Complete active space self-consistent field (CASSCF)[14] calculations augmented with n-electron valence state perturbation theory treatment of the dynamic correlation (NEVPT2)[15] were performed with a def2-SVP basis set. We have chosen minimal active space composed of two oxygen π* orbitals and one π* TEMPO orbital along with five electrons (CAS(5,3)). The CAS wavefunction was averaged over three doublet states, and before calculating NEVPT2 correction, one step of state-specific orbital relaxation was taken for each state to accommodate orbital changes upon the ET process.

Table S8 shows the **π*** and **n(O)** frontier molecular orbitals of the free TEMPO radical and TEMPO anion, as well as the TEMPO anion in complex **2**. Calculations indicate that the reduction of TEMPO radical introduces an excess electron to the π* orbital.[16] The HOMO orbital of the TEMPO anion has the π*(O-N) character (e = 2.25 eV), with the molecular orbital n(O) representing the lone pair of the oxygen being lower in energy (1.01 eV). This picture resembles the situation in complex **2** where the HOMO orbital has a significant π*(O-N) character. However, in this case, the bond order between Ca and O is 0.33 (as shown in Figure 2a in the main text), and the molecular orbital has a significant contribution to the Ca atom. Thus, we conclude that oxidation of **2** (along with the O_2 reduction) can be described as electron removal from the Ca-O bond.

Table S8. The **π*** and **n(O)** frontier molecular orbitals and their energy of the free TEMPO radical, TEMPO anion, and the TEMPO anion in complex **2**.

Orbital	**TEMPO radical**	**TEMPO$^-$**	**Complex 2**
**π***	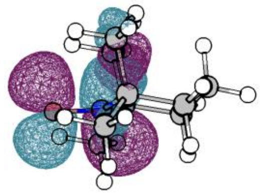<br>e = -2.26 eV<br>(LUMO of the β set)	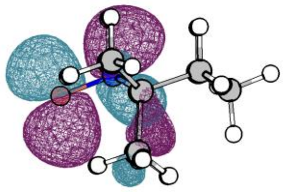<br>e = 2.25 eV<br>(HOMO)	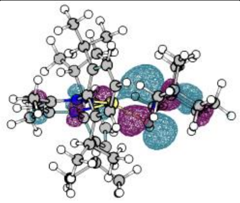<br>e = -4.16 eV<br>(HOMO)
**n(O)**	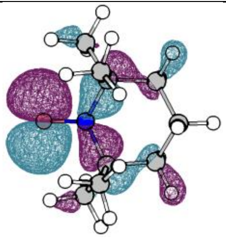<br>e = -5.47 eV<br>(HOMO of the β set)	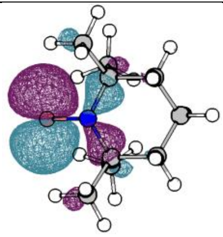<br>e = 1.01 eV<br>(HOMO-1)	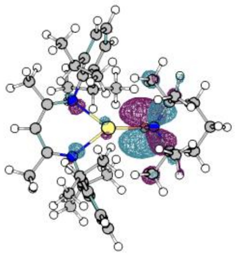<br>e = -4.56 eV<br>(HOMO-2)

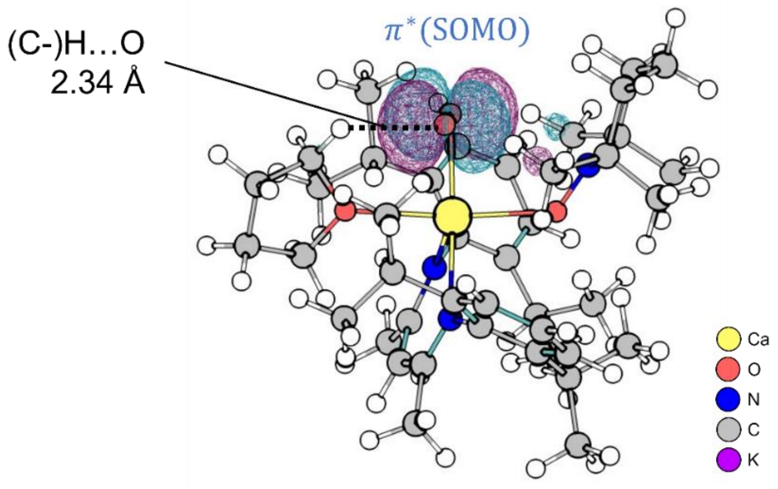

Figure S12. Proximity effect between a H atom from THF and a superoxide moiety in the **[2][$O_2^{\cdot -}$]** structure. The H…O distance of 2.34 Å is highlighted (black dotted line), which is less than the sum of van der Waals radii of H and O (1.2 Å and 1.52 Å, respectively). The singly occupied molecular orbital (SOMO) is drawn at an ±0.03 a.u. isosurface and its nodal planes are found perpendicular to the plane formed by two oxygen atoms and the proximal hydrogen atom. Such orientation facilitates the direct H atom abstraction from THF.

XYZ structures of models

[2]

C	2.4243974	1.0087833	-2.2757274
Ca	1.9761345	1.4947288	1.3355835
C	3.6180495	1.3812347	-1.5996931
N	3.7234409	1.6186700	-0.2859201
H	2.5480980	0.8955495	-3.3620824
N	0.7618870	0.6076032	-0.5181382
C	1.1808739	0.5225546	-1.7874293
C	-0.4186149	-0.0751746	-0.1125966
C	-1.6416961	0.6483279	0.0332042
C	-0.3516345	-1.4455927	0.2728109
C	-2.7862430	-0.0328284	0.4839920
C	-1.5242119	-2.0816182	0.7250522
C	-2.7386517	-1.3935436	0.8172144
H	-3.7355628	0.5121297	0.5876994
H	-1.4785644	-3.1413035	1.0212240
H	-3.6454346	-1.9097180	1.1672284
C	5.0096015	1.7327956	0.3101847
C	5.7388434	0.5544543	0.6445663
C	5.5104216	3.0143765	0.6923505
C	6.9763230	0.6848742	1.3033255
C	6.7530060	3.0936713	1.3461638
C	7.4925704	1.9406102	1.6437033
H	7.5419585	-0.2229169	1.5650510
H	7.1488988	4.0767804	1.6394771
H	8.4625675	2.0216690	2.1572721
C	0.3325763	-0.2183766	-2.8114956
H	0.4922667	0.1824980	-3.8303143
H	0.6264878	-1.2884118	-2.8279683
H	-0.7449213	-0.1878547	-2.5666804
C	4.8695181	1.4078633	-2.4663972
H	5.6271478	2.1178381	-2.0853071
H	5.3444468	0.4042862	-2.4641730
H	4.6257984	1.6564498	-3.5166683

[2][$O_2^{\bullet -}$]

C	2.4072987	1.0136735	-2.1328745
Ca	1.8583266	2.0731283	1.3618821
C	3.5917951	1.4813379	-1.5013143
N	3.6804970	1.8758177	-0.2273335
H	2.5278276	0.7886822	-3.2035178
N	0.7845927	0.7844063	-0.3205277
C	1.1810440	0.5633836	-1.5802712
C	-0.3546321	0.1165269	0.2037774
C	-1.6032572	0.8037725	0.2909517
C	-0.2203646	-1.1993208	0.7383358
C	-2.7148946	0.1247414	0.8235504
C	-1.3636407	-1.8366692	1.2557488
C	-2.6066751	-1.1938752	1.2864751
H	-3.6872441	0.6354617	0.8789682
H	-1.2729590	-2.8589502	1.6542365
H	-3.4894793	-1.7116825	1.6914982
C	4.9438936	2.0356291	0.4036955
C	5.6826845	0.8857673	0.8209853
C	5.4219201	3.3451505	0.7267604
C	6.8693196	1.0712657	1.5548649
C	6.6121219	3.4742502	1.4663660
C	7.3355774	2.3487442	1.8839770
H	7.4375761	0.1866285	1.8821279
H	6.9884048	4.4764562	1.7165834
H	8.2631109	2.4698385	2.4640135
C	0.3138174	-0.2493704	-2.5360817
H	0.1918013	0.2898899	-3.4968600
H	0.8066040	-1.2131873	-2.7755807
H	-0.6839711	-0.4707598	-2.1173247
C	4.8227025	1.4748670	-2.4016769
H	5.7146571	1.8877206	-1.8978536
H	5.0562688	0.4428349	-2.7309199
H	4.6243840	2.0634954	-3.3208947

[2][K^+][$O_2^{\bullet -}$]

C	2.4485723	1.0386728	-2.1418827
Ca	1.5777928	2.3738794	1.1188362
C	3.5609260	1.5752213	-1.4411280
N	3.5312672	1.9889312	-0.1645863
H	2.6458382	0.8077189	-3.1992796
N	0.7107633	0.8360306	-0.4441269
C	1.1848535	0.5861875	-1.6689867
C	-0.4592939	0.2148485	0.0641382
C	-1.6501286	0.9900901	0.2176999
C	-0.4164988	-1.1255784	0.5540361
C	-2.7801014	0.4009398	0.8144273
C	-1.5710972	-1.6666222	1.1515435
C	-2.7500147	-0.9204688	1.2786146
H	-3.7046479	0.9885910	0.9146971
H	-1.5470906	-2.7028581	1.5233428
H	-3.6432500	-1.3682557	1.7398827
C	4.7223465	2.1632549	0.5840212
C	5.5246229	1.0307969	0.9283005
C	5.0480717	3.4487042	1.1185549
C	6.6232747	1.2096825	1.7894473
C	6.1506142	3.5725336	1.9837166
C	6.9393883	2.4656056	2.3226952
H	7.2414520	0.3382658	2.0547199
H	6.4082993	4.5627392	2.3878821
H	7.8023787	2.5825655	2.9952365
C	0.3668508	-0.2253527	-2.6600927
H	0.2897374	0.3134386	-3.6258233
H	0.8632876	-1.1929031	-2.8763264
H	-0.6503917	-0.4359617	-2.2836885
C	4.8531771	1.6628280	-2.2398109
H	5.6331278	2.2341913	-1.7037409
H	5.2581896	0.6536828	-2.4563029
H	4.6618708	2.1446562	-3.2195174

C	0.9611038	-2.2229839	0.2631004
H	1.7514448	-1.5360261	-0.1045209
C	0.9095407	-3.4296377	-0.6927699
C	1.3437681	-2.6556687	1.6918989
H	1.4895016	-1.7763856	2.3533118
H	0.5599669	-3.3050487	2.1350423
H	2.2873149	-3.2377011	1.6823982
H	0.1728278	-4.1858524	-0.3495253
H	1.8990961	-3.9287184	-0.7461251
H	0.6209092	-3.1289652	-1.7202392
C	-1.6907986	2.1417286	-0.2745116
H	-0.7009859	2.5415039	0.0287552
C	-2.7615111	2.8952652	0.5277969
C	-1.8534554	2.4204029	-1.7813612
H	-1.0033581	2.0238543	-2.3658869
H	-2.7819221	1.9515801	-2.1678443
H	-1.9205934	3.5109275	-1.9801630
H	-3.7890313	2.6113840	0.2209002
H	-2.6724305	3.9880891	0.3587579
H	-2.6683854	2.7050133	1.6156677
C	5.1726903	-0.8364608	0.3772083
H	4.2409122	-0.7126132	-0.2108242
C	4.7939945	-1.5211268	1.7045757
C	6.1321469	-1.7112598	-0.4489701
H	6.4215362	-1.2197550	-1.4000766
H	7.0665741	-1.9322302	0.1080121
H	5.6580757	-2.6831666	-0.6962843
H	5.6855041	-1.6441115	2.3547245
H	4.3770391	-2.5312179	1.5156201
H	4.0361792	-0.9387599	2.2684786
C	4.6910177	4.2676335	0.4072329
H	3.6302644	3.9518016	0.4830406
C	4.9040608	4.7853790	-1.0284598
C	4.9153756	5.3968729	1.4240618
H	4.7830797	5.0439344	2.4664368
H	5.9314451	5.8358244	1.3431666
H	4.1976619	6.2244407	1.2449616
H	5.9716655	5.0342047	-1.2024084
H	4.3101491	5.7063681	-1.2108980
H	4.6022870	4.0338536	-1.7815806
H	1.9715470	5.4589669	1.8887529
O	1.3022993	3.7327839	0.9216871
C	1.1750325	4.2624320	-0.4210906
C	0.0945374	5.3625303	-0.3284641
C	-0.1188130	5.5713143	1.1953806
C	1.0584922	4.8239587	1.8254568
H	2.1562910	4.6766201	-0.7336788
H	0.9321401	3.4128460	-1.0866970
H	-0.8446814	5.0490841	-0.8217530
H	0.4380812	6.2883339	-0.8287823
H	-1.0714207	5.1103402	1.5221311
H	-0.1415925	6.6387413	1.4869177
H	0.8596850	4.3852747	2.8216663
N	1.7916712	1.3846648	3.7325047
C	2.9262028	1.8628466	4.5996013
C	2.4671150	3.1197520	5.3674551
C	1.1535749	2.9195705	6.1211629
C	0.0698977	2.4721817	5.1414487
C	0.4406653	1.1894544	4.3683079
H	0.8513320	3.8583418	6.6312787
H	1.2823813	2.1622503	6.9232305
H	-0.8998187	2.3064716	5.6563898

C	1.1294120	-1.9109944	0.7753744
H	1.9019029	-1.1548421	0.5246725
C	1.2145317	-3.0317227	-0.2780910
C	1.4461782	-2.4585675	2.1783397
H	1.4077058	-1.6508333	2.9353118
H	0.7354836	-3.2579607	2.4751666
H	2.4634127	-2.8981394	2.1971789
H	0.4725293	-3.8303372	-0.0669001
H	2.2221281	-3.4968124	-0.2750435
H	1.0157739	-2.6548673	-1.2993758
C	-1.7279747	2.2442490	-0.2002502
H	-0.7574354	2.7399602	0.0192994
C	-2.8300147	3.0346524	0.5191409
C	-1.9383166	2.3207016	-1.7248024
H	-1.0789201	1.9047378	-2.2799890
H	-2.8431304	1.7517297	-2.0233674
H	-2.0797903	3.3724275	-2.0511144
H	-3.8437646	2.6961716	0.2215521
H	-2.7637471	4.1121660	0.2635606
H	-2.7530396	2.9380767	1.6196928
C	5.2294713	-0.5347105	0.4914079
H	4.2413378	-0.4630138	-0.0060191
C	5.0477471	-1.3940158	1.7551154
C	6.2080647	-1.2188017	-0.4843565
H	6.3793490	-0.6136202	-1.3949049
H	7.1960991	-1.3834252	-0.0049819
H	5.8202044	-2.2103497	-0.7971912
H	6.0126257	-1.5434517	2.2839040
H	4.6640602	-2.3992597	1.4828853
H	4.3358693	-0.9186788	2.4569346
C	4.6821255	4.5818449	0.2213650
H	3.5975792	4.3637178	0.3150621
C	4.9643679	4.8280995	-1.2750837
C	4.9867838	5.8644335	1.0090103
H	4.8638427	5.7294928	2.1000066
H	6.0235479	6.2178170	0.8292974
H	4.3108579	6.6827939	0.6857300
H	6.0496115	4.9874434	-1.4437904
H	4.4292116	5.7318132	-1.6365189
H	4.6486816	3.9729189	-1.8983400
H	1.8914996	6.0786568	0.6521105
O	1.3048921	4.1215015	0.1989844
C	1.2985456	4.1869448	-1.2508063
C	0.3482474	5.3500453	-1.6201639
C	-0.1059402	5.9220734	-0.2555647
C	0.9840827	5.4305712	0.6946472
H	2.3345891	4.3682313	-1.6012731
H	0.9875146	3.1922471	-1.6176367
H	-0.5104677	5.0035544	-2.2258792
H	0.8849276	6.1154665	-2.2145766
H	-1.0801451	5.4890214	0.0477047
H	-0.2077580	7.0247002	-0.2585212
H	0.6712465	5.3116448	1.7468593
N	0.9287700	3.9795316	4.1338364
C	2.2629762	3.9922396	4.8213095
C	2.3190033	5.2496934	5.7164949
C	1.1023831	5.3847953	6.6333323
C	-0.1747712	5.4426201	5.7961960
C	-0.3616197	4.2330810	4.8527873
H	1.1909820	6.3015797	7.2515037
H	1.0609068	4.5370541	7.3500622
H	-1.0747850	5.5191526	6.4410811

C	0.8419936	-1.9813519	0.4520398
H	1.6444066	-1.3441476	0.0269114
C	0.6333974	-3.1839724	-0.4878581
C	1.3100677	-2.4488058	1.8405725
H	1.5452138	-1.5774224	2.4818687
H	0.5340403	-3.0587599	2.3482038
H	2.2205901	-3.0751593	1.7622331
H	-0.1153524	-3.8918270	-0.0746439
H	1.5809935	-3.7440323	-0.6273912
H	0.2714281	-2.8657725	-1.4840633
C	-1.7168756	2.4187831	-0.3099619
H	-0.6764913	2.7883063	-0.3980586
C	-2.4623980	3.3745461	0.6335567
C	-2.3071357	2.4502473	-1.7326761
H	-1.7177274	1.8155779	-2.4225851
H	-3.3506929	2.0747008	-1.7385000
H	-2.3147480	3.4829850	-2.1410174
H	-3.5383413	3.1214863	0.7237906
H	-2.4088797	4.4155720	0.2560612
H	-2.0167089	3.3604613	1.6483053
C	5.1726179	-0.3755633	0.4568056
H	4.3841935	-0.2941671	-0.3160461
C	4.5642370	-1.1818471	1.6180207
C	6.3695742	-1.1083453	-0.1730452
H	6.8283951	-0.5114889	-0.9863271
H	7.1629873	-1.3217052	0.5724117
H	6.0497847	-2.0811179	-0.5987739
H	5.2881024	-1.2891505	2.4526336
H	4.2780745	-2.1995159	1.2843980
H	3.6561297	-0.6818144	2.0085285
C	4.2726047	4.6859194	0.6815151
H	3.2491745	4.3594306	0.4100187
C	4.8973647	5.2760571	-0.5994114
C	4.1365134	5.7563298	1.7745208
H	3.6971494	5.3313949	2.6977627
H	5.1116078	6.2211003	2.0297963
H	3.4696109	6.5739047	1.4320619
H	5.9379142	5.6109298	-0.4101456
H	4.3187463	6.1506188	-0.9635877
H	4.9312708	4.5223077	-1.4105792
H	1.4453854	6.2592382	0.1680415
O	1.0713861	4.2586022	-0.3208029
C	1.4336127	4.2646143	-1.7257102
C	0.5323009	5.3237446	-2.3982000
C	-0.2370606	5.9771012	-1.2234842
C	0.5817187	5.5738850	0.0024642
H	2.5103435	4.5212546	-1.8106578
H	1.3025589	3.2323325	-2.1010708
H	-0.1604272	4.8679977	-3.1315329
H	1.1467786	6.0660274	-2.9431361
H	-1.2591568	5.5563294	-1.1450548
H	-0.3330273	7.0751728	-1.3253229
H	0.0099802	5.4942858	0.9450005
N	1.5477289	1.0633003	4.2034443
C	2.8220738	1.2021191	4.9935092
C	2.5140373	1.9740018	6.2954618
C	1.2778822	1.4701669	7.0379675
C	0.0593465	1.5417954	6.1202186
C	0.2142004	0.7262769	4.8181200
H	1.1075061	2.0781244	7.9503048
H	1.4315572	0.4290998	7.3905830
H	-0.8543107	1.1839909	6.6380167

H	-0.1035209	3.2859343	4.4016700
O	2.1792853	0.1966421	3.0518605
C	-0.5609403	1.0089192	3.2150414
H	-0.3785511	0.0716714	2.6573137
H	-1.6005540	0.9759636	3.5951800
H	-0.5030281	1.8675567	2.5102645
C	0.3751623	-0.0631269	5.2640846
H	0.8542088	0.0855702	6.2495497
H	-0.6792569	-0.3529912	5.4442346
H	0.8907443	-0.8917768	4.7429485
H	3.2834629	3.4296950	6.0526521
H	2.3390881	3.9472587	4.6345029
C	4.0595275	2.2687879	3.6423330
H	4.8929492	2.7478566	4.1918919
H	4.4720982	1.3983909	3.0996986
H	3.6952910	3.0144956	2.9030423
C	3.4566077	0.7676917	5.5440300
H	2.8091713	0.6127081	6.4266911
H	3.5135323	-0.1808472	4.9768550
H	4.4689024	1.0300252	5.9114663

H	-0.1525240	6.3637607	5.1735173
O	0.8345345	3.3308677	3.0180471
C	-1.4244258	4.5726405	3.7976893
H	-1.6091503	3.7141306	3.1293981
H	-2.3695634	4.8419461	4.3080539
H	-1.1099535	5.4342718	3.1759023
C	-0.7943771	2.9568877	5.6093506
H	-0.1681698	2.7598523	6.4979664
H	-1.8442140	3.0617649	5.9485827
H	-0.7275287	2.0800009	4.9363786
H	3.2602713	5.2104711	6.3023917
H	2.3861733	6.1458892	5.0609564
C	3.3473834	4.0765188	3.7369799
H	4.3109166	4.3543398	4.2058229
H	3.5189580	3.1011179	3.2408109
H	3.0960033	4.8439952	2.9815245
C	2.4725499	2.7016689	5.6479784
H	1.8961932	2.7083856	6.5910316
H	2.1844394	1.8113980	5.0592028
H	3.5457745	2.6115189	5.9080575
O	1.7495891	0.6095734	3.1899075
O	3.0284329	0.7774455	2.8565485

H	-0.1358469	2.6027409	5.8467535
O	1.6642072	0.8977867	2.9334225
C	-0.8806027	1.1101425	3.8166245
H	-0.8473012	0.4799135	2.9103835
H	-1.8687822	0.9693801	4.2941519
H	-0.7783927	2.1677923	3.5069387
C	0.1253338	-0.7917928	5.0906426
H	0.8038384	-1.1301576	5.8940717
H	-0.9106277	-1.0422498	5.3914475
H	0.3558305	-1.3551692	4.1680200
H	3.4164337	1.9249934	6.9377553
H	2.3730642	3.0488357	6.0431354
C	3.8264005	1.9974645	4.1501281
H	4.7286318	2.1949653	4.7589464
H	4.1502217	1.4452626	3.2498253
H	3.3972230	2.9642458	3.8280572
C	3.3993573	-0.2020912	5.2791519
H	2.8099809	-0.7677978	6.0233965
H	3.4451452	-0.7895341	4.3434557
H	4.4313739	-0.0989988	5.6685347
O	1.6629700	3.9942116	2.8165563
O	0.3509885	3.7918204	2.5671903
K	0.5050503	5.2670334	4.7355132
H	-1.9763830	7.2401615	3.8752049
O	0.0391806	7.6376836	3.7071879
C	0.8654271	8.7842806	3.9548109
C	0.1313336	9.5918354	5.0491675
C	-1.3374584	9.0872833	4.9566325
C	-1.3130879	8.1187985	3.7597558
H	1.8677725	8.4169933	4.2497073
H	0.9702433	9.3788591	3.0183393
H	0.2189179	10.6814431	4.8753561
H	0.5576987	9.3859073	6.0512637
H	-1.6247323	8.5468305	5.8807302
H	-1.5644222	8.6402474	2.8068048
H	-2.0668075	9.9071252	4.8122127
H	-2.4509889	3.7360554	3.9384086
O	-2.0535088	5.1182168	5.4210589
C	-2.5996732	5.4329611	6.7195589
C	-3.5575012	4.2710230	7.0927950
C	-3.4732416	3.3172992	5.8785571
C	-2.9891121	4.2418348	4.7627295
H	-1.7463872	5.5498138	7.4186302
H	-3.1392325	6.4051039	6.6727828
H	-4.5890704	4.6487615	7.2318796
H	-3.2620352	3.7716652	8.0351304
H	-2.7206082	2.5232465	6.0513340
H	-3.8287283	4.8325930	4.3267899
H	-4.4374428	2.8263145	5.6449000
H	4.3100788	6.3318039	4.9314704
O	2.6237423	5.9726356	6.1320085
C	2.6156287	6.7847993	7.3305480
C	4.0830066	7.1047746	7.6386545
C	4.8105501	5.8968645	7.0258029
C	3.9898700	5.6607176	5.7603202
H	2.0001457	7.6921243	7.1483418
H	2.1370017	6.2044400	8.1513301
H	4.2713025	7.2356312	8.7215058
H	4.3930518	8.0390625	7.1263674
H	5.8808168	6.0870306	6.8158521
H	4.0174288	4.6188070	5.3866611
H	4.7445343	5.0160148	7.6982032

[2a][$O_2^{\bullet-}$][2]

C	1.9383157	1.6759570	-1.8797959
Ca	1.8453586	3.0181535	1.5842193
C	3.1868202	2.1173684	-1.3760613
N	3.3578180	2.5617580	-0.1285527
H	1.9405578	1.3846094	-2.9396528
N	0.4753529	1.7704484	0.0797729
C	0.7143333	1.4444009	-1.1986983
C	-0.7152392	1.3438438	0.7218874
C	-1.7430897	2.3000574	0.9953907
C	-0.8365977	0.0083898	1.2128082
C	-2.8937142	1.8825764	1.6897321
C	-2.0099116	-0.3608744	1.8984272
C	-3.0407084	0.5590415	2.1286448
H	-3.6917526	2.6109022	1.8922050
H	-2.1116658	-1.3929277	2.2699157
H	-3.9525592	0.2495040	2.6614995
C	4.6211764	2.8582347	0.4302235
C	5.4718547	1.8040582	0.8855817
C	4.9570338	4.2222044	0.6925626
C	6.6521704	2.1376347	1.5729399
C	6.1465147	4.5023603	1.3912186
C	6.9925168	3.4744966	1.8260275
H	7.3173237	1.3365012	1.9275995
H	6.4129042	5.5484457	1.5989265
H	7.9184595	3.7145759	2.3702070
C	-0.3602762	0.7364902	-2.0108551
H	-0.2996030	1.0237215	-3.0776724
H	-0.2184236	-0.3629785	-1.9589930
H	-1.3767348	0.9516425	-1.6315670
C	4.3765376	2.0350534	-2.3188381
H	5.1600649	2.7683010	-2.0490142
H	4.8436876	1.0280641	-2.2712867
H	4.0649366	2.2019038	-3.3675168
C	0.2948893	-1.0055747	1.0724511
H	1.1127639	-0.5128760	0.5094251
C	-0.1354144	-2.2604382	0.2916022
C	0.8483245	-1.3896513	2.4589171
H	1.1953628	-0.4917505	3.0062428
H	0.0715111	-1.8892835	3.0748161
H	1.7010314	-2.0929285	2.3648853
H	-0.9134563	-2.8306190	0.8406455
H	0.7263658	-2.9414957	0.1336119
H	-0.5537204	-2.0015764	-0.7010712
C	-1.5928982	3.7480105	0.5398173
H	-0.5099491	3.9954838	0.5876341
C	-2.3201348	4.7465999	1.4492424
C	-2.0119798	3.9272860	-0.9315314
H	-1.4045599	3.2953849	-1.6068939
H	-3.0789364	3.6545302	-1.0721201
H	-1.8808148	4.9828769	-1.2462316
H	-3.4232788	4.6883382	1.3436129
H	-2.0173322	5.7807248	1.2051624
H	-2.0734083	4.5834826	2.5161937
C	5.0489962	0.3494168	0.7053583
H	4.4492884	0.2827119	-0.2237495
C	4.1169138	-0.0767648	1.8554497
C	6.2275630	-0.6242943	0.5655246
H	6.9282344	-0.3027323	-0.2309628
H	6.8064454	-0.7141923	1.5080737
H	5.8610248	-1.6399007	0.3130914

[2a][$O_2^{\bullet-}$][KCl][2]

C	-4.8183937	11.9682730	-3.2635831
Ca	-4.0175918	9.0848349	-1.1334959
C	-3.4521163	11.9035801	-2.8825470
N	-2.8929739	10.8602722	-2.2536194
H	-5.1210131	12.9143839	-3.7319460
N	-5.6564280	9.7345871	-2.7448179
C	-5.8042818	10.9431784	-3.3000215
C	-6.5854967	8.7020551	-3.0570281
C	-7.7353306	8.4766388	-2.2489399
C	-6.3008732	7.8312740	-4.1515956
C	-8.6083685	7.4215384	-2.5764414
C	-7.2124507	6.8031820	-4.4535148
C	-8.3639883	6.5967292	-3.6810382
H	-9.5013927	7.2480150	-1.9554963
H	-7.0115486	6.1386543	-5.3076323
H	-9.0629017	5.7858617	-3.9344538
C	-1.4785649	10.7881517	-2.1399167
C	-0.7358707	10.1268025	-3.1649864
C	-0.8100923	11.2981690	-0.9895645
C	0.6640388	10.0611310	-3.0487943
C	0.5901269	11.1764511	-0.9024316
C	1.3309858	10.5818563	-1.9313205
H	1.2450012	9.5756196	-3.8476126
H	1.1104202	11.5719378	-0.0160278
H	2.4272021	10.5139868	-1.8585776
C	-7.0973304	11.2705219	-4.0279361
H	-7.0655490	12.2686293	-4.5008247
H	-7.3160407	10.5113831	-4.8049822
H	-7.9549263	11.2391983	-3.3250696
C	-2.5954173	13.1159563	-3.2107798
H	-2.2560391	13.6118862	-2.2782371
H	-1.6732591	12.8170714	-3.7471827
H	-3.1428160	13.8549212	-3.8236100
C	-5.0367419	8.0211348	-4.9828234
H	-4.3623643	8.6652272	-4.3814544
C	-5.3244835	8.7640780	-6.2999100
C	-4.3040375	6.6967400	-5.2395155
H	-4.0267235	6.2235592	-4.2792469
H	-4.9211499	5.9804553	-5.8192565
H	-3.3752111	6.8733918	-5.8170291
H	-6.0312525	8.1893488	-6.9360427
H	-4.3886291	8.9121596	-6.8787328
H	-5.7649952	9.7622639	-6.1194921
C	-8.0078500	9.3406018	-1.0251595
H	-7.2544321	10.1523016	-1.0399270
C	-7.7941846	8.5323095	0.2669642
C	-9.4022877	9.9886100	-1.0466305
H	-9.5639299	10.5732919	-1.9742815
H	-10.2083797	9.2272656	-0.9877965
H	-9.5310037	10.6736804	-0.1830519
H	-8.5234347	7.6996563	0.3446171
H	-7.9144847	9.1720458	1.1661805
H	-6.7831614	8.0794800	0.2951159
C	-1.4474214	9.5097036	-4.3646725
H	-2.5086685	9.3903956	-4.0662561
C	-0.9026113	8.1145777	-4.7114118
C	-1.4183139	10.4412298	-5.5906907
H	-1.8964468	11.4160355	-5.3757769
H	-0.3756302	10.6368455	-5.9192591
H	-1.9635886	9.9829494	-6.4420580

H	4.6271139	0.0222685	2.8362237
H	3.7906651	-1.1299139	1.7403413
H	3.1968607	0.5402173	1.8758367
C	4.0589081	5.3459970	0.1860277
H	3.0155484	4.9582931	0.1678936
C	4.3972193	5.7058466	-1.2741902
C	4.0667341	6.5870311	1.0849482
H	3.8386964	6.3223745	2.1346147
H	5.0408939	7.1177864	1.0526208
H	3.2922978	7.3033368	0.7526399
H	5.4421054	6.0720306	-1.3520313
H	3.7234854	6.5055991	-1.6446414
H	4.2915692	4.8321331	-1.9454763
H	2.7364022	2.8629287	4.7101582
O	1.3219245	1.8293247	3.5619611
C	-0.0510869	2.0547655	4.0179394
C	0.0672759	2.4063330	5.4997150
C	1.3474855	1.6665405	5.9188106
C	2.2344193	1.8702598	4.6930071
H	-0.4977686	2.8685795	3.4126747
H	-0.6271561	1.1270734	3.8251832
H	-0.8278496	2.1008255	6.0745507
H	0.1964805	3.4996448	5.6211186
H	1.1470316	0.5860103	6.0759670
H	1.8045296	2.0713869	6.8422216
H	2.9933570	1.0782162	4.5393799
O	1.1345944	5.1034645	2.0742250
O	2.0430782	4.7907550	3.0156307
C	-0.9670847	10.4339746	7.5961215
Ca	-0.6834614	10.2872362	3.9301909
C	-2.0094397	9.6949798	6.9745276
N	-2.1317911	9.4839170	5.6577187
H	-1.0692799	10.5127280	8.6880295
N	0.6328446	10.9495572	5.8122529
C	0.2964236	10.8652937	7.1044215
C	1.9784561	11.2344249	5.4468535
C	2.3459472	12.5649549	5.0777973
C	2.9210430	10.1730532	5.3262156
C	3.6700582	12.8185728	4.6782515
C	4.2342502	10.4786650	4.9188219
C	4.6184449	11.7883239	4.6125099
H	3.9667956	13.8401987	4.4008152
H	4.9683009	9.6630105	4.8271452
H	5.6508872	12.0055571	4.2988851
C	-3.0423763	8.5051927	5.1762104
C	-2.6799183	7.1270085	5.2151170
C	-4.2532585	8.9063462	4.5345815
C	-3.5520736	6.1776730	4.6493126
C	-5.0942040	7.9210846	3.9870458
C	-4.7557495	6.5617524	4.0466215
H	-3.2736137	5.1126876	4.6719619
H	-6.0299097	8.2220970	3.4941812
H	-5.4236135	5.8050369	3.6083951
C	1.3377335	11.1593883	8.1761905
H	0.8648701	11.5357079	9.1030669
H	1.8706697	10.2204906	8.4354255
H	2.1045569	11.8781092	7.8339317
C	-2.9896053	9.0337038	7.9343698
H	-3.9765808	8.8559453	7.4682552
H	-2.5991558	8.0419009	8.2444066
H	-3.1182172	9.6381794	8.8523034
C	2.5346177	8.7158120	5.5603724
H	0.1670520	8.1392857	-5.0023964
H	-1.4611249	7.6883158	-5.5677026
H	-1.0206122	7.4272209	-3.8522254
C	-1.5847958	11.9813483	0.1311025
H	-2.6289915	12.0883082	-0.2280910
C	-1.0446114	13.3878131	0.4441942
C	-1.6218074	11.1092861	1.3988512
H	-2.1134917	10.1356047	1.2077435
H	-0.5970218	10.8992459	1.7694148
H	-2.1778891	11.6120953	2.2167053
H	-0.0208664	13.3482745	0.8679193
H	-1.6876579	13.9039170	1.1876140
H	-1.0027193	14.0161963	-0.4676437
H	-4.1444948	9.9857729	2.3133020
O	-5.0171365	10.5407880	0.5247166
C	-5.4835128	11.8561437	0.1419414
C	-5.1112318	12.7408540	1.3251600
C	-5.4127743	11.8133803	2.5169303
C	-5.1077601	10.4059561	1.9678148
H	-5.0077627	12.1196063	-0.8228423
H	-6.5859497	11.8279931	-0.0147590
H	-5.6878506	13.6857072	1.3528439
H	-4.0334895	12.9969211	1.2824668
H	-6.4809162	11.8945813	2.8025050
H	-4.8098382	12.0513854	3.4142021
H	-5.9124733	9.6814505	2.2116615
K	-2.3377342	4.8088432	-1.9322007
H	-4.6375159	6.3116511	0.5702278
O	-3.3955433	7.9479937	0.9366896
C	-2.0822021	7.3729440	1.1590876
C	-2.1934263	6.7086787	2.5281111
C	-3.6439602	6.1700922	2.5344955
C	-4.3519019	6.9671675	1.4198463
H	-1.8544733	6.6250331	0.3738180
H	-1.3498194	8.1994569	1.0953253
H	-2.0260622	7.4485782	3.3364340
H	-1.4669680	5.8797044	2.6262778
H	-3.6245643	5.0939408	2.2727765
H	-5.2458414	7.5248715	1.7627761
H	-4.1350768	6.3040554	3.5179786
H	-6.5530221	3.7108538	-3.1255831
O	-4.6790101	3.4934818	-2.2164161
C	-5.0463971	2.7025207	-1.0758383
C	-5.8166587	3.6742016	-0.1659056
C	-6.4307505	4.7014033	-1.1554864
C	-5.8250759	4.3007340	-2.5183725
H	-5.6843030	1.8453720	-1.4013639
H	-4.1161194	2.3290337	-0.6043355
H	-5.0883739	4.1546366	0.5186560
H	-6.5769365	3.1574644	0.4516159
H	-7.5367766	4.6706807	-1.1827338
H	-5.4888224	5.1663750	-3.1160789
H	-6.1390992	5.7380392	-0.8997454
H	-0.4186263	7.9030348	-1.4919930
O	-0.0090639	5.9189284	-1.1172100
C	0.8898403	5.2622773	-0.2143635
C	1.2829212	6.3468906	0.7928752
C	1.2841885	7.6379334	-0.0610935
C	0.4648997	7.2579245	-1.3204361
H	0.3297029	4.4166933	0.2310649
H	1.7832445	4.8820971	-0.7673547
H	2.2585709	6.1429056	1.2757180

H	1.4520556	8.6950054	5.8060383
C	3.2910229	8.1020063	6.7534293
C	2.7512248	7.8893297	4.2795628
H	2.1235376	8.2672187	3.4470556
H	3.8141867	7.9255010	3.9628109
H	2.4897254	6.8234809	4.4273420
H	4.3822350	8.0517622	6.5542027
H	2.9401054	7.0665830	6.9431446
H	3.1495869	8.6896544	7.6830139
C	1.3058200	13.6812773	5.0907125
H	0.3471998	13.1988717	4.8075827
C	1.5887978	14.7882542	4.0645499
C	1.1157089	14.2891700	6.4937838
H	0.7440402	13.5428913	7.2193371
H	2.0756429	14.6901467	6.8795657
H	0.3885578	15.1288897	6.4696044
H	2.4904801	15.3792421	4.3264614
H	0.7423592	15.5045246	4.0261808
H	1.7381972	14.3747067	3.0477435
C	-1.3367884	6.6751890	5.7790464
H	-0.8510630	7.5576662	6.2412006
C	-0.4236698	6.1897651	4.6414324
C	-1.4955963	5.6070981	6.8749013
H	-2.1493933	5.9580591	7.6989006
H	-1.9408063	4.6736388	6.4729260
H	-0.5089536	5.3440312	7.3094020
H	-0.8606996	5.3110067	4.1253419
H	0.5733827	5.8871733	5.0173718
H	-0.2576210	6.9653498	3.8662172
C	-4.6101320	10.3859987	4.4451456
H	-3.6438598	10.9220193	4.3460392
C	-5.2709854	10.8941063	5.7408063
C	-5.4717796	10.7438310	3.2254625
H	-5.0229810	10.3737704	2.2822661
H	-6.4953710	10.3226886	3.3012902
H	-5.5863286	11.8448499	3.1452150
H	-6.2053516	10.3330343	5.9510673
H	-5.5343786	11.9696425	5.6573544
H	-4.5998752	10.7762498	6.6116181
H	-3.4538758	12.8799191	2.6230156
O	-1.8359018	12.3603495	3.8382868
C	-2.2285126	13.0675482	5.0399608
C	-2.2418046	14.5631006	4.6507056
C	-2.1120827	14.5628262	3.1032412
C	-2.3665719	13.1015769	2.7265723
H	-3.2352475	12.7133121	5.3447866
H	-1.5095211	12.7885901	5.8332807
H	-1.4034756	15.1107740	5.1203583
H	-3.1804552	15.0446130	4.9867117
H	-1.0874847	14.8523109	2.7986521
H	-2.8213379	15.2536990	2.6083183
H	-1.8430471	12.7485761	1.8183025
N	-0.2704576	9.9085952	1.5852258
C	-1.2839552	9.1952031	0.7324349
C	-1.8521050	10.1980487	-0.2931789
C	-0.7674209	10.9100514	-1.1003618
C	0.1949390	11.6176233	-0.1475867
C	0.8283898	10.6698686	0.8903167
H	-1.2221975	11.6383746	-1.8044826
H	-0.2152180	10.1795020	-1.7288081
H	1.0097962	12.1297547	-0.7011313
H	-0.3592619	12.4136518	0.3987669
H	0.5225272	6.4000259	1.5963196
H	0.8384817	8.4985284	0.4728435
H	1.0954960	7.3017991	-2.2369636
H	2.3121550	7.9380833	-0.3426559
C	1.0497837	-3.9962413	4.3970958
Ca	0.6582680	-1.6161895	1.6270087
C	-0.0800403	-4.2556339	3.5741239
N	-0.4308469	-3.5397522	2.5006713
H	1.1366462	-4.6699843	5.2607708
N	2.3067559	-2.2376097	3.2384261
C	2.1931250	-3.1819460	4.1842241
C	3.5798107	-1.6625248	2.9744180
C	3.8667932	-0.3358911	3.4185359
C	4.5182880	-2.3605998	2.1596093
C	5.1089465	0.2363352	3.0921540
C	5.7453124	-1.7420046	1.8526864
C	6.0521129	-0.4602626	2.3232901
H	5.3441262	1.2539382	3.4356067
H	6.4724446	-2.2762687	1.2206623
H	7.0185488	0.0057861	2.0773339
C	-1.4703342	-3.9963463	1.6449505
C	-1.1862421	-4.9472122	0.6216179
C	-2.7664900	-3.4017031	1.7311149
C	-2.2216811	-5.3313086	-0.2526729
C	-3.7684533	-3.8221077	0.8383359
C	-3.5081658	-4.7899212	-0.1423117
H	-2.0085355	-6.0665480	-1.0442332
H	-4.7711197	-3.3757023	0.9029023
H	-4.3054278	-5.1071431	-0.8319570
C	3.3890825	-3.4997506	5.0707037
H	3.0654832	-3.9412352	6.0320246
H	4.0428653	-4.2416224	4.5662996
H	4.0164162	-2.6092758	5.2644290
C	-0.8687602	-5.5066581	3.9354228
H	-1.9266530	-5.4403751	3.6201983
H	-0.4334790	-6.3849356	3.4136112
H	-0.8204619	-5.7101578	5.0219271
C	4.1947965	-3.7231291	1.5529724
H	3.2270872	-4.0578833	1.9782068
C	5.2514079	-4.7858141	1.9031054
C	4.0132035	-3.5989817	0.0276747
H	3.1834715	-2.9089630	-0.2294247
H	4.9407896	-3.2207280	-0.4508170
H	3.7834559	-4.5876729	-0.4188649
H	6.2368464	-4.5468066	1.4511936
H	4.9446876	-5.7809726	1.5205350
H	5.4006092	-4.8717551	2.9985790
C	2.8273937	0.4468655	4.2159722
H	1.8403939	0.1591375	3.7960007
C	2.9631948	1.9689154	4.0714762
C	2.8193741	0.0452807	5.7032478
H	2.5805004	-1.0262576	5.8352233
H	3.8099922	0.2342313	6.1666403
H	2.0658342	0.6329148	6.2683088
H	3.8900313	2.3518272	4.5475452
H	2.1153166	2.4800901	4.5698773
H	2.9749216	2.2806553	3.0082364
C	0.2173621	-5.5088734	0.4179399
H	0.8634578	-5.0846348	1.2144083
C	0.7909047	-5.0534169	-0.9380316
C	0.2548973	-7.0421564	0.5522660
H	-0.1659074	-7.3817226	1.5202798

O	0.2940328	8.9926908	2.5130868
C	1.5226590	11.5119729	1.9737641
H	2.0401071	10.8710704	2.7114651
H	2.2793040	12.1855323	1.5258013
H	0.7888731	12.1564073	2.5052976
C	1.8842143	9.7425189	0.2583063
H	1.5724036	9.3370492	-0.7217238
H	2.8417817	10.2805879	0.1109058
H	2.0528995	8.8995230	0.9551776
H	-2.5620834	9.6609562	-0.9570189
H	-2.4483532	10.9594065	0.2588103
C	-2.4200353	8.7705561	1.6784070
H	-3.2661838	8.3300202	1.1158630
H	-2.0813227	8.0194378	2.4162497
H	-2.8206144	9.6532586	2.2212044
C	-0.7121380	7.9325221	0.0582917
H	-0.0568648	8.1621508	-0.8012669
H	-0.1289076	7.3620860	0.8072534
H	-1.5382271	7.2971689	-0.3187075
H	-0.3266087	-7.5347778	-0.2548015
H	1.2979988	-7.4145528	0.4833948
H	0.1626980	-5.4174448	-1.7780161
H	1.8119028	-5.4614138	-1.0861412
H	0.8495767	-3.9469619	-0.9974461
C	-3.0387475	-2.3153667	2.7662229
H	-2.0777199	-1.7741728	2.8889922
C	-3.3993233	-2.9035604	4.1442702
C	-4.0957107	-1.2917708	2.3240970
H	-3.8683078	-0.8713707	1.3245600
H	-5.1124044	-1.7357197	2.2835144
H	-4.1406490	-0.4507724	3.0462348
H	-4.3017674	-3.5457411	4.0708593
H	-3.6183853	-2.0955235	4.8744330
H	-2.5743082	-3.5136400	4.5561265
H	-2.1614067	0.9972892	2.3396964
O	-0.4429826	-0.0367501	2.9704154
C	-0.7045401	-0.1063173	4.3891172
C	-0.7426177	1.3601117	4.8759598
C	-0.7951407	2.2037419	3.5745880
C	-1.0737194	1.1752851	2.4800118
H	-1.6812545	-0.6125583	4.5471628
H	0.0876579	-0.7330435	4.8416503
H	0.1527415	1.6070277	5.4783779
H	-1.6278099	1.5306654	5.5189351
H	0.1782275	2.6961068	3.3839746
H	-1.5674972	2.9929856	3.5767595
H	-0.6352578	1.4171275	1.4967623
N	0.7059631	-0.5101085	-0.5693423
C	-0.5228969	-0.4988105	-1.4450432
C	-1.0170740	0.9533919	-1.6158404
C	0.0858958	1.9084463	-2.0659849
C	1.2196491	1.8650703	-1.0469941
C	1.8185527	0.4590727	-0.8645198
H	-0.3000114	2.9452191	-2.1359975
H	0.4581174	1.6454510	-3.0802023
H	2.0411312	2.5588096	-1.3205350
H	0.8183742	2.2104426	-0.0701878
O	1.2185092	-1.8165269	-0.4623003
C	2.7239931	0.4793519	0.3775789
H	3.2393822	-0.4866035	0.5311164
H	3.5061797	1.2581895	0.2832785
H	2.1334569	0.7307943	1.2863269
C	2.6648513	0.0303058	-2.0787287
H	2.1541225	0.2184389	-3.0410099
H	3.6245698	0.5843700	-2.0908054
H	2.8746246	-1.0534898	-2.0000917
H	-1.8662284	0.9452146	-2.3320622
H	-1.4239133	1.3309134	-0.6532994
C	-1.5962199	-1.2985281	-0.6882211
H	-2.5698112	-1.2475767	-1.2143454
H	-1.3235053	-2.3671050	-0.5951867
H	-1.7551607	-0.8712236	0.3252365
C	-0.2714374	-1.1764457	-2.8063780
H	0.3003598	-0.5337780	-3.5010681
H	0.2967579	-2.1114928	-2.6431676
H	-1.2360016	-1.4247374	-3.2930382
Cl	-2.1067433	3.6443829	0.7494864
O	-2.7589468	7.4247502	-2.0532129
O	-4.0098084	6.9148255	-1.8490725

10. References.

(1) Stender, M.; Wright, R. J.; Eichler, B. E.; Prust, J.; Olmstead, M. M.; Roesky, H. W.; Power, P. P. The Synthesis and Structure of Lithium Derivatives of the Sterically Encumbered *β*-Diketiminate Ligand [{(2,6-$Pr^i{}_2H_3C_6$)N(CH_3)C}$_2$CH]$^-$, and a Modified Synthesis of the Aminoimine Precursor. *J. Chem. Soc. Dalton Trans.* **2001**, *2001* (23), 3465–3469. https://doi.org/10.1039/b103149j.

(2) Neufeld, R.; Stalke, D. Accurate Molecular Weight Determination of Small Molecules via DOSY-NMR by Using External Calibration Curves with Normalized Diffusion Coefficients. *Chem. Sci.* **2015**, *6* (6), 3354–3364. https://doi.org/10.1039/C5SC00670H.

(3) Kreyenschmidt, A.-K.; Bachmann, S.; Niklas, T.; Stalke, D. Molecular Weight Estimation of Molecules Incorporating Heavier Elements from Van-Der-Waals Corrected ECC-DOSY. *ChemistrySelect* **2017**, *2* (24), 6957–6960. https://doi.org/10.1002/slct.201701497.

(4) CrysAlisPro (Oxford Diffraction & Agilent Technologies UK Ltd, Yarnton, England).

(5) Sheldrick, G. M. A Short History of SHELX. *Acta Crystallogr. A* **2008**, *64* (1), 112–122. https://doi.org/10.1107/S0108767307043930.

(6) Dolomanov, O. V.; Bourhis, L. J.; Gildea, R. J.; Howard, J. A. K.; Puschmann, H. *OLEX2* : A Complete Structure Solution, Refinement and Analysis Program. *J. Appl. Crystallogr.* **2009**, *42* (2), 339–341. https://doi.org/10.1107/S0021889808042726.

(7) Becke, A. D. Density-Functional Exchange-Energy Approximation with Correct Asymptotic Behavior. *Phys. Rev. A* **1988**, *38* (6), 3098–3100. https://doi.org/10.1103/PhysRevA.38.3098.

(8) Grimme, S.; Antony, J.; Ehrlich, S.; Krieg, H. A Consistent and Accurate *Ab Initio* Parametrization of Density Functional Dispersion Correction (DFT-D) for the 94 Elements H-Pu. *J. Chem. Phys.* **2010**, *132* (15), 154104. https://doi.org/10.1063/1.3382344.

(9) Weigend, F.; Ahlrichs, R. Balanced Basis Sets of Split Valence, Triple Zeta Valence and Quadruple Zeta Valence Quality for H to Rn: Design and Assessment of Accuracy. *Phys. Chem. Chem. Phys.* **2005**, *7* (18), 3297. https://doi.org/10.1039/b508541a.

(10) TURBOMOLE v.7.6 (University of Karlsruhe and Forschungszentrum Karlsruhe, 1989–2007).

(11) Grimme, S.; Hansen, A.; Ehlert, S.; Mewes, J.-M. R^2SCAN-3c: A "Swiss Army Knife" Composite Electronic-Structure Method. *J. Chem. Phys.* **2021**, *154* (6), 064103. https://doi.org/10.1063/5.0040021.

(12) Marenich, A. V.; Cramer, C. J.; Truhlar, D. G. Universal Solvation Model Based on Solute Electron Density and on a Continuum Model of the Solvent Defined by the Bulk Dielectric Constant and Atomic Surface Tensions. *J. Phys. Chem. B* **2009**, *113* (18), 6378–6396. https://doi.org/10.1021/jp810292n.

(13) Neese, F. Software Update: The ORCA Program System—Version 5.0. *WIREs Comput. Mol. Sci.* **2022**, *12* (5). https://doi.org/10.1002/wcms.1606.

(14) Roos, B. O.; Taylor, P. R.; Sigbahn, P. E. M. A Complete Active Space SCF Method (CASSCF) Using a Density Matrix Formulated Super-CI Approach. *Chem. Phys.* **1980**, *48* (2), 157–173. https://doi.org/10.1016/0301-0104(80)80045-0.

(15) Angeli, C.; Cimiraglia, R.; Evangelisti, S.; Leininger, T.; Malrieu, J.-P. Introduction of *n*-Electron Valence States for Multireference Perturbation Theory. *J. Chem. Phys.* **2001**, *114* (23), 10252–10264. https://doi.org/10.1063/1.1361246.

(16) Kubala, D.; Regeta, K.; Janečková, R.; Fedor, J.; Grimme, S.; Hansen, A.; Nesvadba, P.; Allan, M. The Electronic Structure of TEMPO, Its Cation and Anion. *Mol. Phys.* **2013**, *111* (14–15), 2033–2040. https://doi.org/10.1080/00268976.2013.781695.
